# Pharmacologic glycoengineering of Fcγ receptor IIIa enhances force-resistant IgG-FcγR interactions and anti-tumor antibody efficacy

**DOI:** 10.1016/j.immuni.2026.03.028

**Published:** 2026-04-24

**Authors:** Bowie Yik-Ling Cheng, Raquel M. Centeio, David Kung-Chun Chiu, Casey L. Kiyohara, Ella Herzog, Rony Dahan, Wendy E. Thomas, Taia T. Wang

**Affiliations:** 1 Institute for Immunity, Transplantation and Infection, Stanford University School of Medicine, Stanford, CA 94305, USA; 2 Department of Pathology, Stanford University School of Medicine, Stanford, CA 94305, USA; 3 Department of Bioengineering, University of Washington, 3720 15th Ave NE, Foege N430P, Box 355061, Seattle, WA, USA; 4 Department of Systems Immunology, Weizmann Institute of Science, 7610001 Rehovot, Israel; 5 Department of Medicine, Division of Infectious Diseases, Department of Microbiology and Immunology, Stanford University School of Medicine, Stanford, CA 94305, USA; 6 Lead contact

## Abstract

Therapeutic monoclonal antibodies (mAbs) are central to cancer treatment but often show incomplete efficacy. We show that transient pharmacologic inhibition of complex N-glycans in host cells (“glycoengineering”) enhances the *in vivo* activity of multiple depleting mAbs, including mAbs already engineered for heightened potency. In preclinical models, glycoengineering improved α-CD20-mediated tumor clearance and survival through FcγRIIIa- and natural killer (NK) cell-dependent pathways. In B16-F10 melanoma, glycoengineering similarly enhanced anti-CD25 depletion of intratumoral regulatory T cells (Tregs). Notably, glycoengineering produced minimal changes in equilibrium binding affinity but markedly increased the mechanical durability of IgG-FcγRIIIa interactions under physiological shear stress. These results establish antibody effector function as a mechano-immunological process in which IgG-FcγR interactions can be tuned for resilience to physiological forces, thereby moving beyond the current affinity-centric paradigm in mAb engineering. Integrating mechanobiology into therapeutic development may enable mAbs optimized for the dynamic forces of human physiology, which provides a route to enhance next-generation immunotherapies.

## In brief

Therapeutic monoclonal antibodies are central to cancer treatment but often show incomplete efficacy. Cheng et al. demonstrate that transient host-cell glycoengineering to reduce N-glycan trimming strengthens IgG-FcγRIIIa bond resilience under vascular flow forces and potentiates therapeutic antibody efficacy in lymphoma and melanoma models. This study establishes force durability as an optimization parameter for therapeutic antibodies beyond the affinity-centric paradigm.

## Graphical Abstract

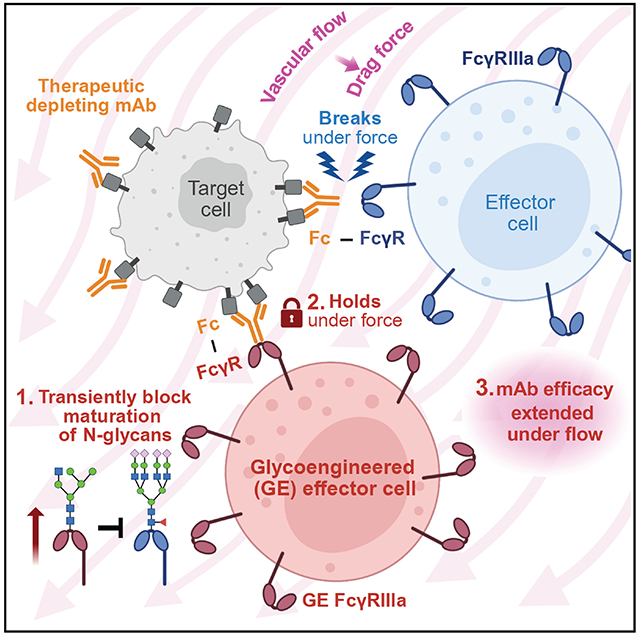

## INTRODUCTION

Monoclonal antibodies (mAbs) have transformed the treatment of cancer and autoimmune diseases by enabling highly specific targeting of pathological cells. Hundreds of FDA-approved mAbs now exist, with rituximab, an α-CD20 mAb that depletes B cells through antibody-dependent cellular cytotoxicity (ADCC) and other mechanisms, remaining an important therapy for non-Hodgkin lymphoma, chronic lymphocytic leukemia, and rheumatoid arthritis.^1^ Similarly, trastuzumab, which targets HER2-overexpressing cells, has markedly improved outcomes in breast and other cancers.^2^ More recently, clinical trials have explored regulatory T cell (Treg)-depleting antibodies (e.g., the α-CD25 antibody RG6292) to enhance antitumor immunity.^3^ These therapeutic antibodies exemplify the remarkable success of mAb-based cancer immunotherapy; how to further optimize their efficacy and broaden clinical responses remains an important area of investigation.

The efficacy of many clinical mAbs relies on their engagement of Fcγ receptors (FcγRs) expressed on immune effector cells.^4,5^ Among these, FcγRIIIa, a low-affinity activating receptor primarily expressed on natural killer (NK) cells and select myeloid populations, drives ADCC by bridging mAb-coated target cells to cytotoxic effectors.^6^ Beyond direct killing, FcγRIIIa engagement can also promote inflammatory mediator release and enhance recruitment of immune cells to sites of antibody deposition.^7–9^ However, several factors can limit the efficacy of cytotoxic mAbs, including low FcγRIIIa expression on effector cells and the FcγRIIIa-F158 polymorphism, which reduces IgG-binding affinity and has been linked to less favorable clinical responses.^10^

The persistent issues of heterogeneity in clinical responses and acquired resistance provide compelling evidence that our current understanding of depleting mAb mechanisms remains incomplete. Critical knowledge gaps likely exist in multiple dimensions, including regulatory factors governing effector cell activation, alternative cytolytic mechanisms beyond classical ADCC, biophysical determinants of IgG-FcγR engagement under physiologic forces, and system-level host factors influencing treatment outcomes. Addressing these gaps may reveal new opportunities to enhance mAb efficacy and overcome current therapeutic limitations.

Current strategies to enhance mAb-mediated effector functions focus predominantly on increasing the binding affinity between IgG and specific FcγRs, as exemplified by afucosylated antibodies that enhance FcγRIIIa binding by over 20-fold.^11^ A complementary opportunity may come from a deeper understanding of the mechanobiology of FcγR-IgG interactions, as mechanical forces *in vivo* could shape receptor engagement and downstream effector responses in ways not captured by equilibrium affinity measurements. While immune receptors, including T cell receptor-MHC interactions and selectin-ligand interactions, are well established as mechanosensitive,^12,13^ the functional impact of mechanical forces on IgG-FcγR interactions remains largely unexplored.

Standard affinity-based assays such as bead-based binding assays and enzyme-linked immunosorbent assays (ELISAs) notably lack the physiologically relevant fluid flow conditions or cytoskeletal contractions that generate forces on molecular bonds *in vivo*. Thus, widely used *in vitro* systems may miss force-dependent features of IgG-FcγR interactions that only emerge under physiologic mechanical stress. As a consequence, mechanobiological determinants of antibody effector function are not routinely measured or incorporated into current evaluation and optimization pipelines, even though antibodies operate in dynamic environments where they must withstand physiological forces including circulatory shear stress and contractile forces. Accordingly, a key question is whether current antibody engineering approaches miss an important determinant of IgG-FcγR interactions by failing to account for mechanobiology.

Glycosylation, the enzymatic attachment of carbohydrate molecules (glycans) to proteins, profoundly shapes protein interactions and functions. In N-linked glycosylation, a high-mannose glycan is added to asparagine (N) residues and trimmed in the Golgi by mannosidases, after which several glycosyltransferases add sugars like N-acetyl-glucosamine, galactose, and sialic acid to form complex glycans. Under-processed glycans typically retain high-mannose forms, while complex glycans can be highly sialylated, reflecting extensive modifications. Complex N-glycans can suppress mAb-mediated immune responses through multiple mechanisms, including engagement of inhibitory glycan-binding proteins such as sialic acid-binding immunoglobulin-like lectin (Siglec) receptors on effector cells, and by reducing IgG-FcγRIIIa interactions leading to reduced immune cell activation.^14–21^

To investigate whether transient inhibition of complex glycan formation could enhance mAb efficacy, we employed swainsonine, an orally available α-mannosidase II inhibitor with established clinical safety profiles, to glycoengineered (GE) host cells *in vivo*.^22,23^ Using FcγR-humanized mouse models, we found that GE enhanced mAb-mediated depletion of endogenous CD20^+^ B cells and CD4^+^ T cells. In cancer models, GE improved antitumor efficacy of both conventional and afucosylated α-CD20 antibodies and potentiated Fc-engineered α-CD25-mediated depletion of intratumoral Tregs. Notably, this enhanced activity occurred without substantial increases in IgG-FcγRIIIa binding affinity under static conditions, leading us to hypothesize that physiologic force might be required to reveal changes in the IgG-FcγR interaction. Using a vascular shear stress simulation platform,^24,25^ we discovered that GE improves the mechanical durability of FcγRIIIa-IgG interactions, enhancing their resistance to dissociation under physiological shear conditions. These findings reveal an important mechanobiological dimension of antibody effector function that has been overlooked in current therapeutic development approaches.

## RESULTS

### GE of FcγRIIIa potentiates mAb-mediated cytotoxicity and cell depletion

**Figure 1.**
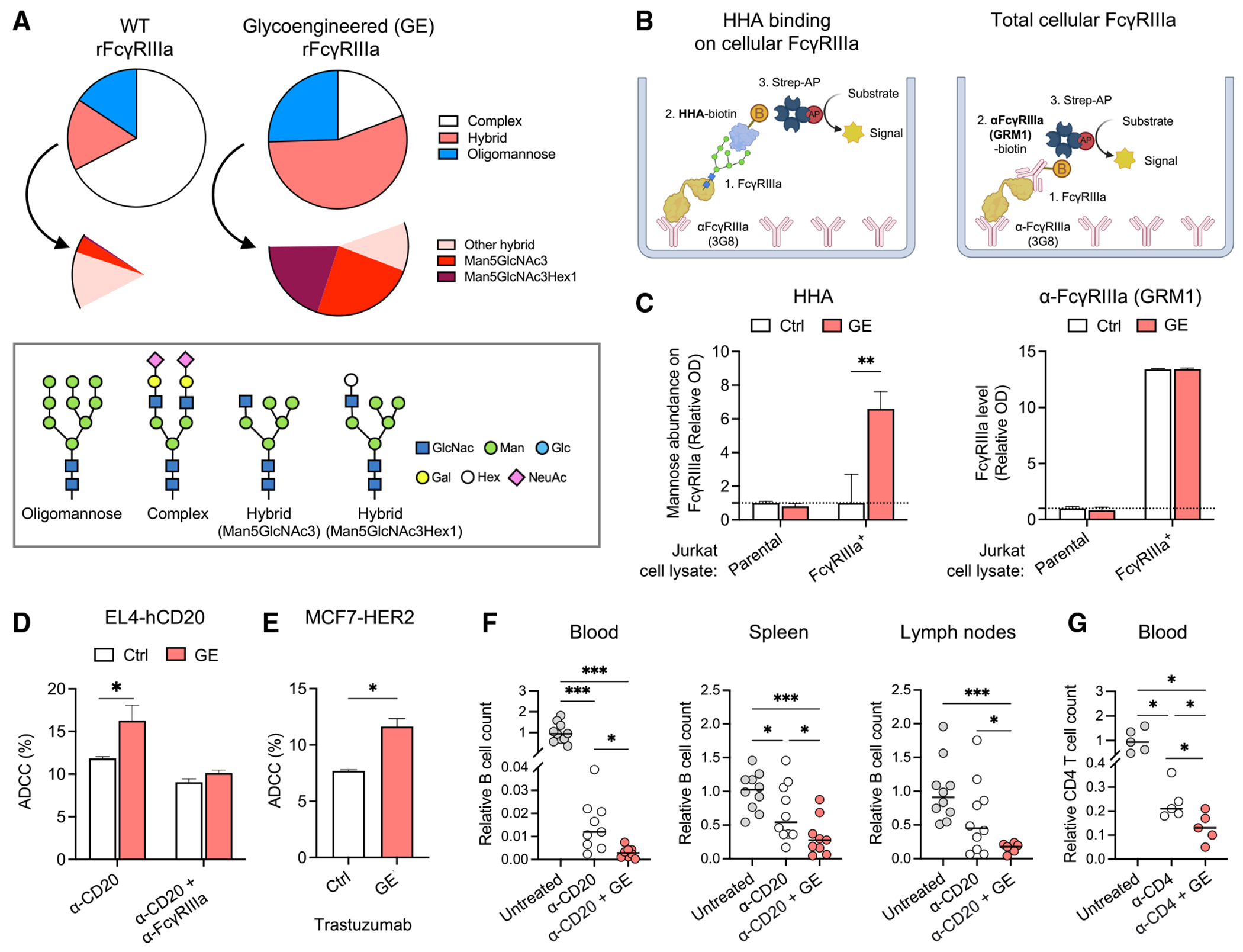
Glycoengineering of FcγRIIIa potentiates mAb-mediated cytotoxicity and cell depletion (A) Proportion of specific glycans on WT and swainsonine-treated (GE) recombinant FcγRIIIa (rFcγRIIIa) (top). Examples of glycoform structures (bottom). (B) Schematic of FcγRIIIa-specific lectin-ELISA: HHA binding (left) and FcγRIIIa protein (right). (C) Mannose-specific lectin HHA binding (left) and total FcγRIIIa protein (right) on FcγRIIIa protein captured by α-FcγRIIIa from the cell lysates of FcγRIIIa^+^ and FcγRIIIa^−^ Jurkat cells with or without swainsonine treatment (GE). (D) α-CD20-mediated ADCC response of untreated (Ctrl) and GE-PBMCs pretreated with either isotype control or FcγRIIIa-blocking antibody, against EL4-hCD20 cells. (E) Trastuzumab-mediated ADCC response of untreated (Ctrl) and GE-PBMCs against HER2^+^ MCF-7 cells. (F) B cells were quantified from blood, spleen, and lymph nodes of mice treated with α-CD20 alone or in combination with swainsonine (GE). (G) T cells were quantified from blood of mice treated with α-CD4 alone or in combination with swainsonine (GE). All mAbs were expressed as human IgG1. Graphs show mean ± SEM in (C)–(E) and median in (F) and (G). Statistical significance was calculated with two-tailed paired Student’s *t* test (C), two-way ANOVA with Tukey’s multiple comparison test (D), and two-tailed unpaired Student’s *t* test (E–G). *p < 0.05, **p < 0.01, ***p < 0.001. See also Figures S1 and S2.

To explore whether GE of host effector cells can enhance mAb-mediated cytotoxicity and target cell depletion, we used the Golgi α-mannosidase II inhibitor swainsonine to suppress N-linked complex glycan formation (Figure S1A). Mammalian cell-expressed FcγRIIIa (rFcγRIIIa) exhibited a distinct glycan profile when produced in the presence of swainsonine, showing enrichment for under-processed, highly mannosylated N-glycans relative to wild-type (WT) rFcγRIIIa (Figure 1A), including at positions N45 and N162, which are modulators of the Fc-FcγRIIIa interaction (Figure S1B).^26,27^ Accordingly, GE effector cells exhibited higher binding by the α1-3, α1-6 mannose-specific hippeastrum hybrid lectin (HHA), alongside reduced binding of erythrina cristagalli lectin (ECL), which binds to galactose residues, commonly found in complex glycan forms (Figure S1C). FcγRIIIa protein isolated from the lysate of GE effector cells also showed higher binding of HHA, while FcγRIIIa expression remained unchanged compared to unmodified cells (Figures 1B and 1C).

To test whether GE human effector cells enhanced mAb-mediated cytotoxicity and target cell clearance, we utilized a lymphoma model in which EL4 murine cells express human CD20 as the target antigen (EL4-hCD20) (Figures S1D and S1E), and α-CD20 IgG, generated from a rituximab-encoding plasmid, serves as the therapeutic mAb.^28^ We selected EL4-hCD20 for these *in vitro* ADCC assays because EL4 target cells do not express FcγRs, ensuring that the Fc domain of anti-CD20 engages FcγRs only on the effector cells (and not on the target cells). By contrast, many B cell lines (and endogenous B cells) express the inhibitory receptor FcγRIIb, the type II Fc receptor CD23, and other IgG-binding receptors that can alter antibody binding/processing and trigger target-cell-intrinsic signaling, complicating attribution of cytotoxicity to effector cell FcγR pathways.^29,30^ Co-culture of α-CD20-opsonized EL4-hCD20 cells with human peripheral blood mononuclear cells (PBMCs) pretreated with FcγR-blocking antibodies revealed that only blockade of FcγRIIIa, but not other FcγRs, abrogated α-CD20-mediated ADCC (Figure S2A).^31,32^ Moreover, GE-PBMCs treated with swainsonine exhibited enhanced α-CD20-mediated ADCC in an FcγRIIIa-dependent manner, underscoring the central role of FcγRIIIa in this system (Figures 1D, S2B, and S2C). Enhanced ADCC was also mediated by GE-PBMCs in a separate model using trastuzumab (α-HER2) to target HER2-expressing MCF-7 breast cancer cells (Figure 1E).

To determine whether GE effector cells also enhanced target cell clearance *in vivo*, we utilized a C57BL/6 mouse model engineered to express human type I FcγRs together with human CD20 (hFcγR/hCD20).^33,34^ Unlike WT C57BL/6 mice, which lack a functional ortholog of human FcγRIIIa on NK cells and display species-specific differences in FcγR expression and function, this humanized model provides a more physiologically relevant system for studying IgG-FcγR interactions *in vivo*. In this setting, treatment with swainsonine resulted in greater B cell and CD4^+^ T cell depletion in response to α-human CD20 or α-murine CD4 mAbs, respectively (Figures 1F, 1G, and S2D–S2F).

### GE potentiates the efficacy of both standard and afucosylated α-CD20

**Figure 2.**
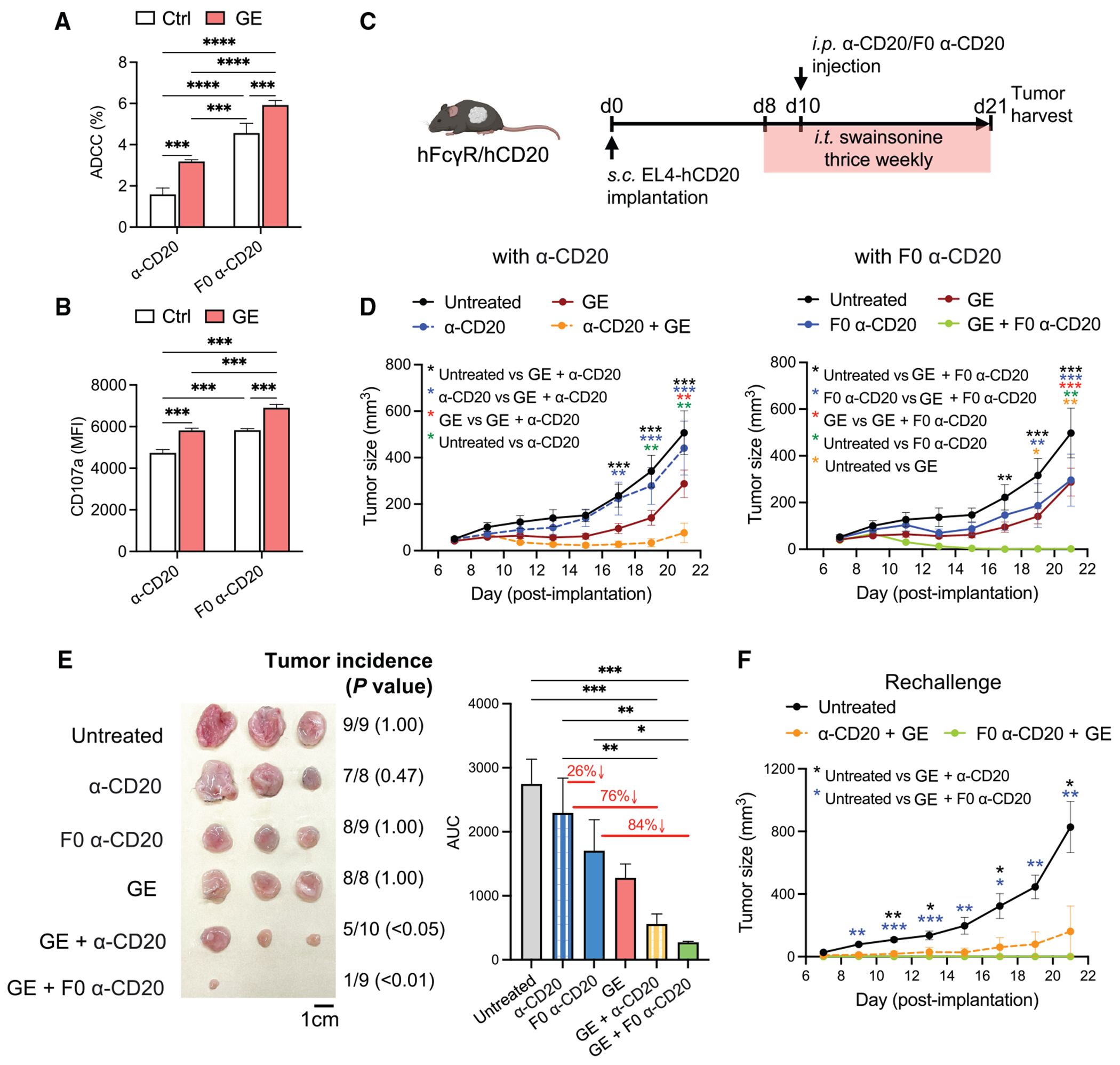
Glycoengineering potentiates the efficacy of both standard and afucosylated mAbs in a murine cancer model (A and B) EL4-hCD20 cells opsonized with α-CD20 or its afucosylated (F0) version were co-incubated with human PBMCs pretreated with swainsonine (GE), followed by detection of (A) ADCC and (B) induction of NK cell degranulation measured by CD107a expression. (C) Schematic diagram of the murine subcutaneous lymphoma model. EL4-hCD20 cells were subcutaneously (s.c.) implanted in hFcγR/hCD20 mice. Swainsonine was given intratumorally (i.t.) thrice per week from day 8 onward, alone or in combination with intraperitoneal (i.p.) injection of α-CD20 or F0 α-CD20 on day 10. (D) Longitudinal assessment of tumor size in all treatment groups. (E) Representative picture of tumors and tumor incidence compared with the control group (left) and area under curve (AUC) of tumor growth. Scale bar represents 1 centimeter. (F) Surviving mice from the combined treatment group were allowed to age, followed by rechallenge of EL4-hCD20 cells via s.c. implantation without further treatment, followed by longitudinal assessment of tumor size. Graphs show mean ± SEM in (A), (B), and (D)–(F). Statistical significance was determined in (A) and (B) by two-way ANOVA with Tukey’s multiple comparison test, in (D) and (F) by one-way ANOVA, and in (E) with Fisher’s exact test. *p < 0.05, **p < 0.01, ***p < 0.001. See also Figures S3 and S4.

We next tested whether GE could potentiate the activity of afucosylated α-CD20, a form of the mAb that is engineered to have higher potency as a consequence of increased affinity for FcγRIIIa (Figure S2G). As expected, afucosylated α-CD20 mediated greater ADCC and NK cell degranulation *in vitro* when compared with fucosylated/WT α-CD20,^35,36^ with GE effector cells further enhancing ADCC and NK cell degranulation mediated by both WT and afucosylated α-CD20 (Figures 2A and 2B). GE of human PBMCs also potentiated the release of inflammatory factors when incubated with afucosylated α-CD20-opsonized tumor cells, including the release of TNF-α, IFN-γ, and C-C motif chemokine ligand 3 (CCL3) (Figure S3A). This enhanced release was dependent on the presence of opsonized tumor cells (Figure S3B).

Consistent with prior publications, afucosylated α-CD20 also provided superior suppression relative to WT α-CD20 in the absence of GE, in a CD20-expressing cancer model (Figures S4A and S4B).^35^ Addition of GE enhanced the antitumor activity of both WT and afucosylated α-CD20, leading to both reduced tumor growth and tumor incidence (Figures 2C–2E). The combination of swainsonine and afucosylated α-CD20 achieved the most pronounced therapeutic effect, with complete tumor regression in 8 of 9 mice (Figure 2E). Importantly, swainsonine administration did not alter CD20 expression on tumor cells or the production of endogenous α-EL4-hCD20 antibodies (Figures S4C and S4D).

In this intratumoral injection setting, GE alone showed some protective activity that was accompanied by a local reduction in cell-surface sialic acids (Figure S4E) and enhanced recognition of GE EL4-hCD20 cells by mannose-binding lectin (MBL) and CD206 (Figure S4F). To test whether the protective activity of swainsonine alone was FcγRIII-dependent, we implanted EL4-hCD20 tumors subcutaneously into hFcγR/hCD20/FcγRIIIa^KO^ mice (which lack FcγRIIIa) and treated with intratumoral swainsonine. In the absence of FcγRIIIa, we still observed a clear trend toward reduced tumor size with swainsonine alone (Figure S4G).

Prior studies have established that FcγRIIIa activity, together with FcγRIIa, is required for α-CD20 therapy to elicit a “vaccinal” antitumor effect in which long-term antitumor immunity is generated following α-CD20 administration.^34^ We hypothesized that the enhanced FcγRIIIa-dependent effector activity achieved through GE might augment this effect by increasing the availability of tumor antigens and α-CD20-containing immune complexes, thereby improving antigen presentation and priming of tumor-specific T cells. To test this hypothesis, we rechallenged all mice that survived tumor implantation with a lethal tumor dose approximately 1 year after the initial treatment. All surviving mice were from the GE groups that had additionally received either WT or afucosylated α-CD20 during the primary challenge (Figures 2C–2E). Mice that received GE during the initial α-CD20 treatment were protected against rechallenge, with those that initially received afucosylated α-CD20 showing complete protection (Figure 2F). Notably, the protection against tumor growth observed 1 year after initial tumor clearance was comparable to that observed during primary tumor implantation when therapeutic mAbs were administered. These findings support the hypothesis that GE enhances the generation of durable antitumor immune memory; however, future studies will be required to define precise mechanisms involved.

### GE potentiates afucosylated α-CD20 in a FcγRIIIa- and NK cell-dependent manner

**Figure 3.**
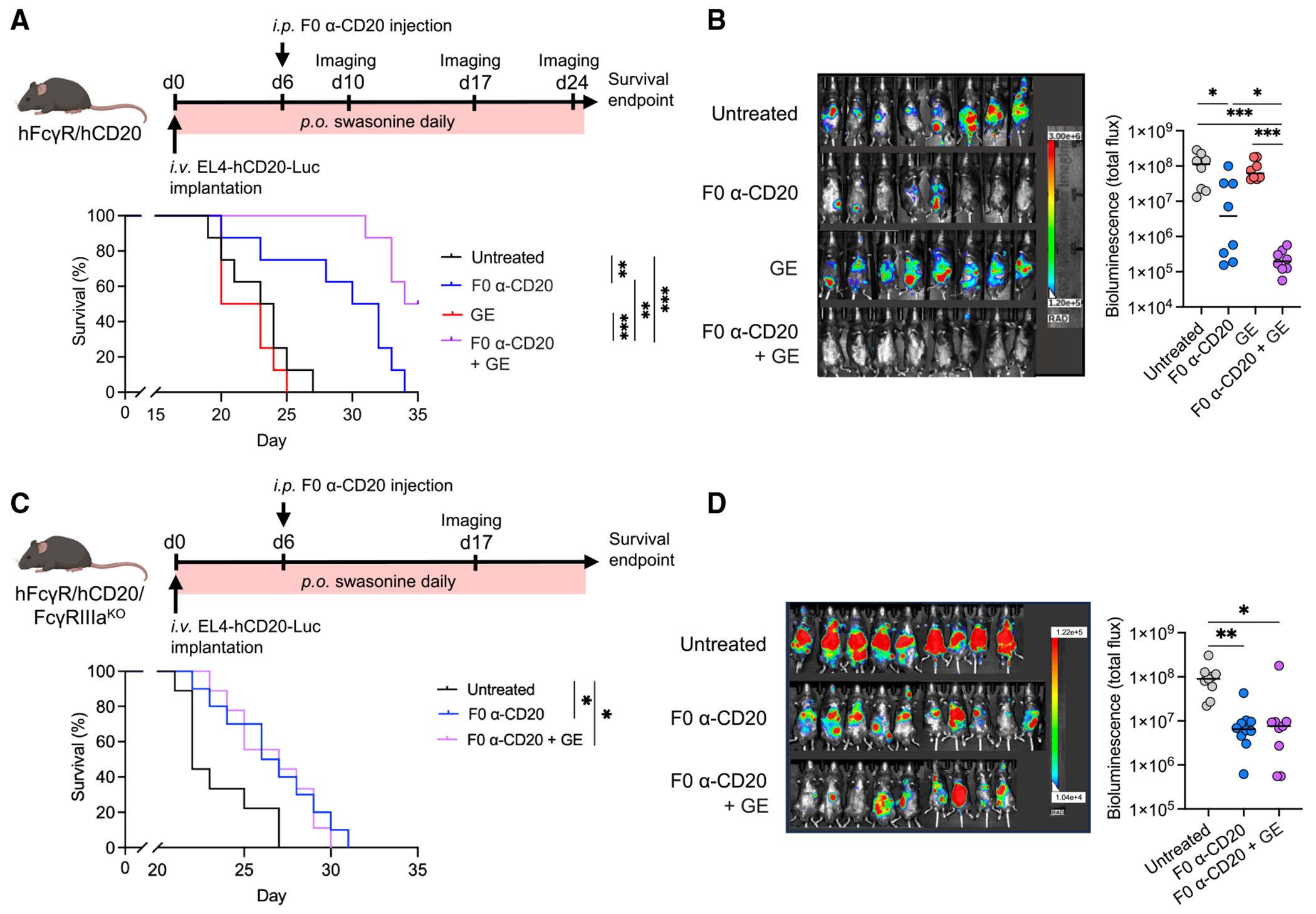
Glycoengineering potentiates afucosylated α-CD20 in a FcγRIIIa-dependent manner (A and B) EL4-hCD20 cells overexpressing luciferase (EL4-hCD20-Luc) were intravenously (i.v.) implanted into hFcγR/hCD20 mice. Swainsonine was given orally (daily) and afucosylated α-CD20 (F0 α-CD20) was i.p. administered on day 6. Survival (A) and cancer burden (B) as determined by measuring bioluminescence emitted from cancer cells on day 17. (C and D) EL4-hCD20-Luc cells were inoculated into hFcγR/hCD20/FcγRIIIa^KO^ mice and the same protocol was followed as in (A). Survival (C) and cancer burden (D) on day 17 are shown. Graphs show the median in (B) and (D). Statistical significance was determined by log-rank Mantel-Cox test in (A) and (C) and Mann-Whitney test in (B) and (D). *p < 0.05, **p < 0.01, ***p < 0.001. See also Figure S5.

Next, we tested the activity of orally administered swainsonine in a disseminated hematologic lymphoma model. We used afucosylated αCD20 in these experiments to determine whether GE could further potentiate a mAb already engineered for enhanced effector function in a second *in vivo* setting. As in the solid tumor model, GE enhanced afucosylated αCD20-mediated cancer cell clearance and improved survival (Figures 3A, 3B, and S5A–S5C). Notably, we observed the antitumor activity of swainsonine alone only when administered intratumorally (Figure 2) and not with oral delivery (Figure 3), suggesting that a high local concentration is required for this standalone effect.

Because swainsonine broadly alters glycosylation beyond FcγRIIIa, we tested whether its effects on α-CD20 activity were FcγRIIIa-dependent. In contrast to FcγRIIIa-expressing mice, GE did not enhance the therapeutic efficacy of afucosylated α-CD20 in FcγRIIIa^KO^ mice, establishing that the *in vivo* enhancement of afucosylated α-CD20 by GE is FcγRIIIa-dependent (Figures 3C and 3D).

**Figure 4.**
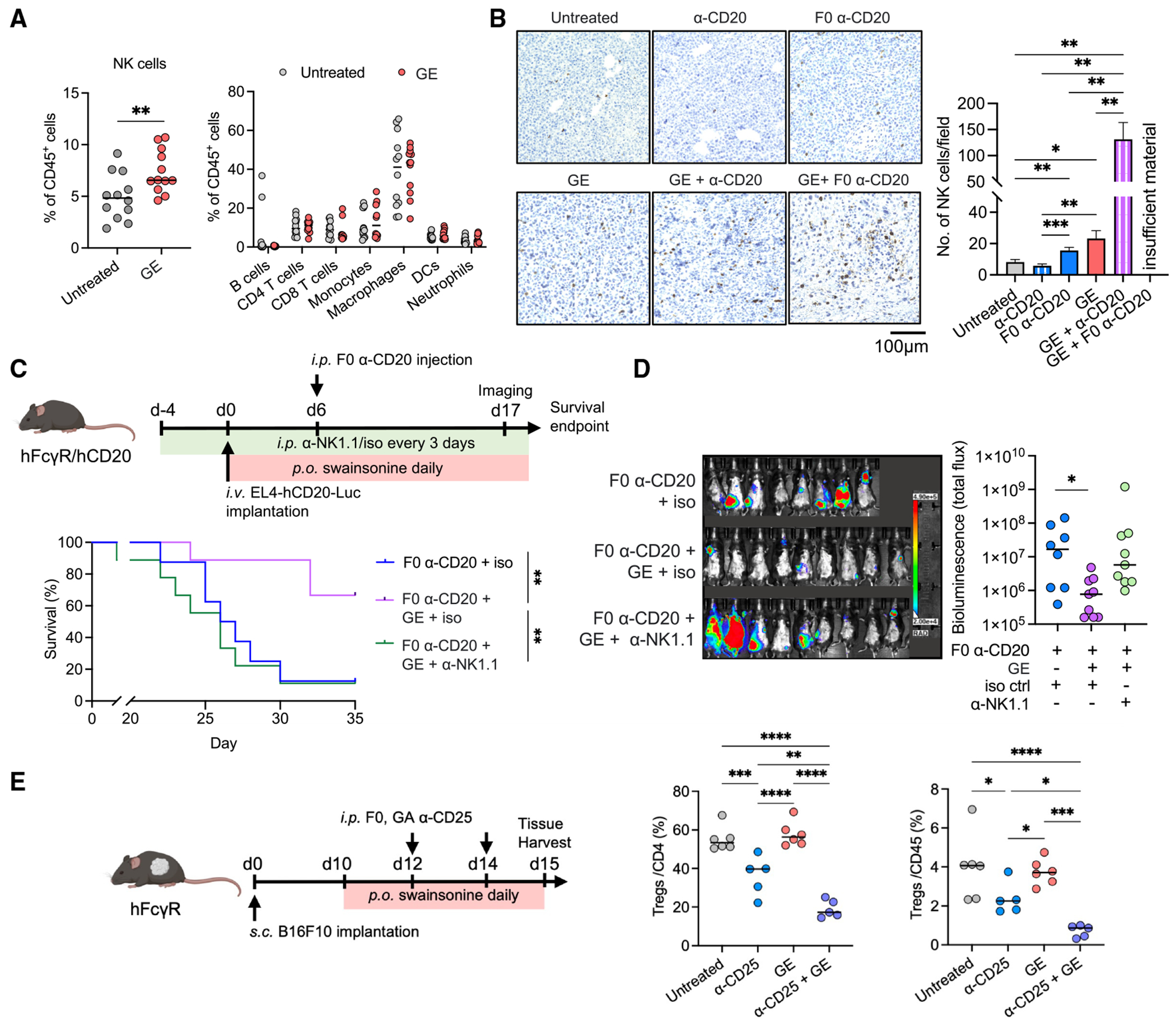
GE potentiates afucosylated α-CD20 in a NK cell-dependent manner (A) Frequencies of immune effector cell populations in the tumor microenvironment (TME) of mice treated with F0 α-CD20, with or without swainsonine-mediated GE. (B) Representative histological images of NK cells (NK1.1 staining) (left) and quantification of NK cells (right) in excised tumors from mice treated under different treatment conditions. (C) Schematic diagram showing cancer model established by i.v. delivery of EL4-hCD20-Luc, followed by a single F0 α-CD20 treatment with daily oral swainsonine administration with either NK cell-depleting antibody (α-NK1.1) or isotype control antibody. (D) Cancer burden as determined by measuring bioluminescence emitted from EL4-hCD20-Luc on day 17. (E) The B16-F10 melanoma model was established by s.c. implantation in hFcγR mice, followed by daily oral swainsonine starting on day 10 and two i.p. doses of α-CD25 (afucosylated, G236A [F0GA]) on days 12 and 14 (left). Quantification of intratumoral Tregs as a fraction of total CD4 T cells (middle) or total CD45^+^ cells (right). Graphs show median in (A) and (D)–(E) and mean ± SEM in (B). Statistical significance was determined by two-tailed unpaired Student’s *t* test in (A), Brown-Forsythe and Welch ANOVA test in (B), log-rank Mantel-Cox test in (C), Mann-Whitney test in (D), and ordinary one-way ANOVA in (E). *p < 0.05, **p < 0.01, ***p < 0.001, ****p < 0.0001. See also Figure S5.

To investigate how GE modulates the tumor microenvironment (TME), we analyzed immune cell dynamics in the solid tumor model. GE led to a selective increase in NK cell frequency within the TME, without detectable changes in other populations including macrophages, monocytes, dendritic cells, T cells (CD4^+^ and CD8^+^), or B cells (Figure 4A). Increased NK cell infiltration in tumors from GE and α-CD20 or afucosylated α-CD20-treated mice was also observed by immunohistochemistry (Figure 4B), supporting a role for NK cells in the enhanced antibody-mediated antitumor efficacy by GE.

Given this increase in intratumoral NK cells, we next tested whether NK cells were important for the enhanced protection conferred by GE in the hematologic lymphoma model. hFcγR/hCD20 mice were pretreated with either an NK cell-depleting antibody (α-NK1.1) or an isotype control prior to intravenous injection of EL4-hCD20 cells (Figures 4C, 4D, and S5D). NK cell depletion completely abolished the therapeutic benefit conferred by GE (Figures 4C and 4D). Notably, NK cell depletion did not reduce the activity of afucosylated α-CD20 alone, which is consistent with prior work^34^ (Figures S5E and S5F), indicating that GE specifically recruits an NK cell-dependent component to antibody efficacy. Together, these findings indicate that GE enhances α-CD20 activity by increasing FcγRIIIa-dependent effector functions and by recruiting NK cell-dependent mechanisms *in vivo*.

### GE enhances Fc-engineered α-CD25 to promote intratumoral Treg depletion

To evaluate the broad applicability of GE across different target cells and tumor types, we tested this approach in the B16-F10 melanoma model using an Fc-engineered α-CD25 depleting antibody. We selected this model because CD25 is highly enriched on intratumoral Tregs, which suppress antitumor immunity, providing a well-defined system to assess how GE impacts the depleting activity of a therapeutic antibody in an immunosuppressive TME. The α-CD25 mAb used in this study was already optimized for enhanced FcγR engagement through two modifications: afucosylation to increase FcγRIIIa binding and a G236A (GA) mutation to boost FcγRIIa affinity.^37^ In FcγR-humanized mice bearing established B16-F10 tumors, oral administration of swainsonine (GE alone) had no effect on Treg frequency, whereas GE enhanced the ability of α-CD25 to deplete intratumoral Tregs by day 15. Thus, consistent with our findings using afucosylated α-CD20, GE can further enhance the activity of Fc-engineered antibodies, improving depletion of immunosuppressive Tregs in an immunologically “cold” tumor.

### GE enhances the force durability of the IgG-FcγRIIIa interaction

**Figure 5.**
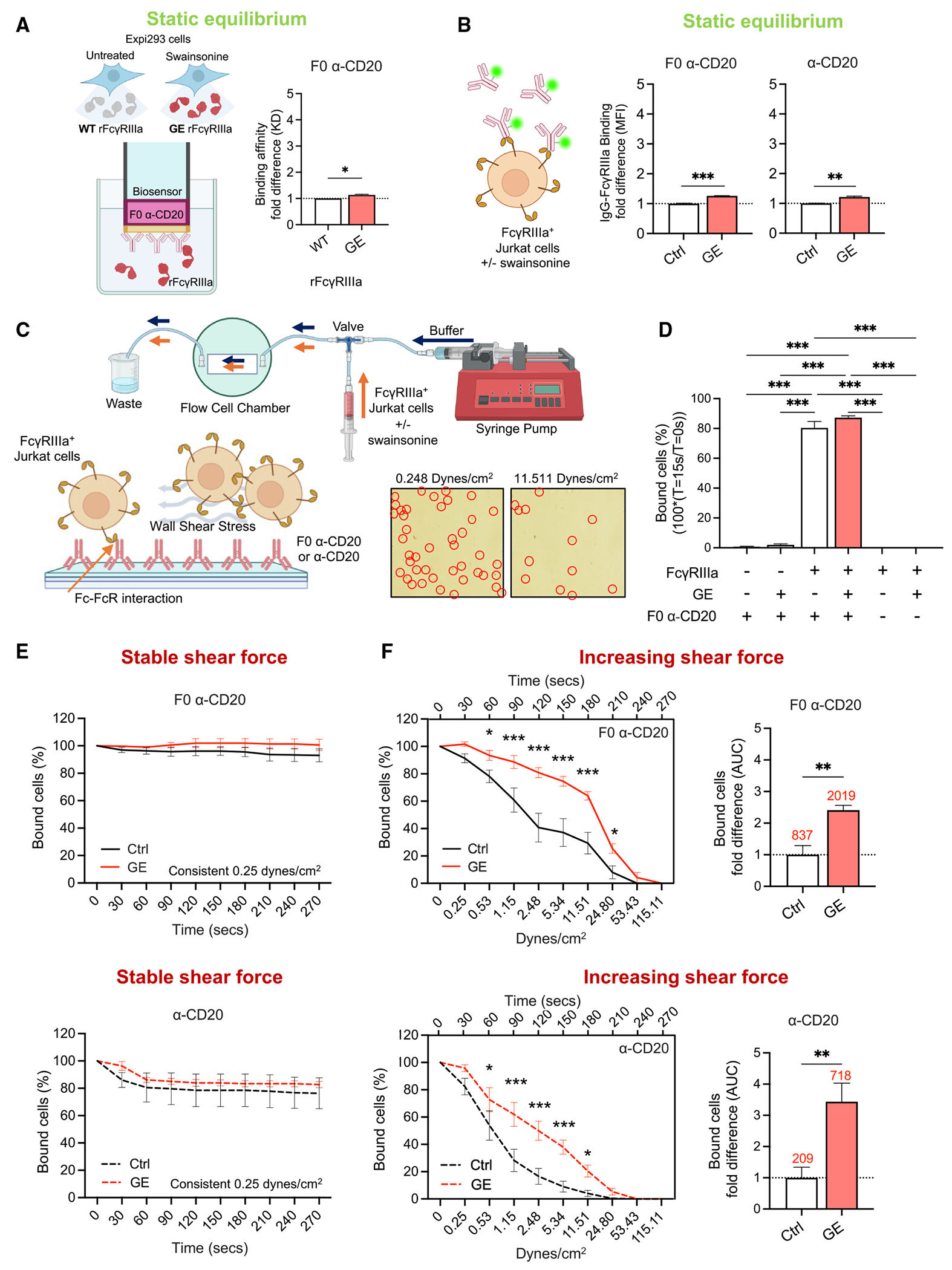
GE enhances the force durability of the IgG-FcγRIIIa interaction (A) Recombinant FcγRIIIa (rFcγRIIIa) was produced in Expi293 cells with or without swainsonine treatment, followed by bio-layer interferometry (BLI) measurement of binding affinity between antibodies immobilized on the BLI probe and rFcγRIIIa in solution. (B) The binding of fluorescently labeled antibodies (α-CD20 or F0 α-CD20) onto the FcγRIIIa^+^ expressing cells. (C) Schematic of the fluidics flow chamber setup. Antibodies (α-CD20 or F0 α-CD20) were immobilized on the chamber surface, and untreated or GE FcγRIIIa^+^ cells were allowed to adhere. Wall shear stress was then either maintained at 0.25 dynes/cm^2^ or increased in ∛10 dyne/cm^2^ increments by progressively raising the buffer flow rate. Cells remaining bound after 30 s at each shear in dynes/cm^2^ were quantified. (D) Controls were performed to determine the dependence of cell binding within the chamber on the presence of antibodies and FcγRIIIa. (E) After initial cell adherence, wall shear stress was maintained at 0.25 dynes/cm^2^, and the number of bound cells was quantified every 30 s. (F) After initial cell adherence, wall shear stress was increased in ∛10 dynes/cm^2^ increments, and the number of bound cells was quantified every 30 s. AUC is presented on the right, with actual AUC in red above each column. Graphs show mean ± SEM in (A), (B), and (D)–(F). Statistical significance was calculated by two-tailed unpaired Student’s *t* test in (A), (B), (E), and (F), and one-way ANOVA in (D). *p < 0.05, **p < 0.01, ***p < 0.001. See also Figure S5.

Throughout our experiments, we observed a discrepancy between the modest effects of GE on *in vitro* binding measurements and its substantial enhancement of mAb efficacy *in vivo*. For context, while afucosylation of α-CD20 increased FcγRIIIa binding affinity by >20-fold and reduced tumor burden by 26% compared to the WT antibody (Figure 2E, right), GE produced only a 1.2-fold affinity increase (Figures 5A and S5G) yet reduced tumor burden by 76%–84% (Figure 2E, right). This enhancement occurred despite minimal changes in cell-based binding assays, suggesting that conventional equilibrium measurements fail to capture the key mechanisms underlying the therapeutic benefit of GE. Notably, GE and afucosylation produced similar improvements in ADCC activity *in vitro* (Figure 2A) despite their vastly different effects on binding, further indicating that affinity alone poorly predicts FcγR-mediated function.

This observation prompted the hypothesis that GE enhances IgG-FcγRIIIa interactions specifically under mechanical forces encountered *in vivo*, which are absent in static assays. To test this, we developed a fluidics platform that allows us to study how cellular drag forces resulting from fluidic shear stress in the circulatory system impact the interaction between IgG and FcγRIIIa.^25^ This platform enables measurement of the binding of FcγRIIIa-expressing cells in suspension to immobilized IgG under fluidics with tunable shear stress (Figure 5C). IgG-FcγR binding in the fluidics chamber was dependent on both the presence of IgG (F0 α-CD20) and expression of FcγRIIIa on the Jurkat cells (Figure 5D). While GE and control FcγRIIIa-expressing cells showed similar binding over time at low shear (0.25 dynes/cm^2^) (Figure 5E), GE cells exhibited substantially greater force resistance as shear increased (Figure 5F), with particular resistance by the afucosylated IgG (F0) at shear present in venous (<10 dynes/cm^2^), as opposed to arterial (>10 dynes/cm^2^) circulation.^38^ This enhanced binding strength in a biomechanical environment (Figure 5F) stands in stark contrast to the minimal change in binding strength in an equilibrium affinity assay (Figures 5A and 5B).

Together, these results demonstrate that the IgG-FcγRIIIa interaction can be modified to enhance resistance to physiological fluid forces, suggesting that the improved activity of depleting mAbs observed with GE *in vivo* involves biomechanical processes, which are not part of the current paradigm.

## DISCUSSION

This study establishes a strategy to enhance mAb therapies that synergizes with established Fc engineering strategies, including afucosylation and G236A mutations, and fundamentally redefines IgG-FcγRIIIa engagement as a force-sensitive process. Our findings suggest that incorporating physiologically relevant forces into the evaluation of IgG-FcγR interactions may reveal determinants of mAb activity that are not apparent from equilibrium measurements alone.

Immune cells must maintain stable attachments to opsonized target cells in the presence of cytoskeletal forces, the dynamic flow environments of the circulatory system, and other physiological forces within tissues. Given our observation that fluid shear impacts the stability of IgG-FcγR interactions, physiological forces warrant consideration when predicting antibody function *in vivo*—a dimension not captured by static binding assays. Static assays neither assess bond kinetics nor the force-dependence of detachment rate constants and thus fail to predict the mechanical strength of molecular interactions under physiologically relevant forces.^39–41^ To address this gap, microfluidic assays such as those used here may improve the prediction of antibody function by quantifying the force resistance of IgG-FcγR interactions, thereby incorporating a key physiological dimension absent from standard binding studies. The enhanced retention of the IgG-FcγR interactions that we observed could reflect the improved mechanical stability of individual bonds, changes in receptor clustering, altered mechanotransduction signaling, changing cell behavior, or a combination of these processes. Related biomechanical principles also contribute to immune synapse formation, including cytoskeletal contractility in T cells, raising the possibility that GE may also enhance FcγR-dependent function in contexts without overt fluid flow by modulating force-bearing cell-cell contacts.

Another notable finding was that GE with swainsonine functionally recruited NK cells in a setting where NK cells were otherwise dispensable for afucosylated αCD20-mediated tumor clearance. Shifting N-glycans away from complex structures and toward under-processed/high-mannose forms was associated with increased NK cell infiltration and a requirement for NK cells in the therapeutic efficacy of afucosylated αCD20, whereas the baseline (complex-glycan-rich) state was associated with an absence of NK cell dependence. This pattern supports the idea that complex-type glycans, which can bear terminal sialic acids, can dampen NK cell recruitment and/or activation, while preventing maturation of N-glycans removes this constraint.^17^ Mechanistically, our data further support a model in which GE enhances FcγRIIIa-dependent interactions on NK cells, promoting more effective degranulation and cytokine production, including chemotactic factors that could amplify NK cell accumulation at sites of antibody deposition. Importantly, the use of humanized FcγR mice was essential for revealing this NK cell-dependent phenotype, as murine NK cells do not express a direct functional equivalent of human FcγRIIIa and rely on distinct FcγR usage. Thus, conventional mouse models may underestimate or misrepresent IgG-dependent NK cell effector functions.

Overall, this study demonstrates that IgG-FcγR affinity is an incomplete predictor of antibody effector function. Instead, antibody-receptor interactions have an orthogonal, force-dependent dimension that can dominate *in vivo* outcomes under physiologically relevant shear and cell-generated forces. This work redefines ADCC as a force-sensitive process and establishes force durability as a tunable parameter that can be modulated independently of static affinity. Although we focused on cancer and endogenous immune cell depletion models, the findings may extend to other disease contexts in which antibodies encounter dynamic mechanical environments. For example, pathogens in shear-prone vasculature or tissues with elevated interstitial pressure may be more effectively targeted by antibodies optimized for force durability. In contrast to antibody engineering approaches that require molecule-specific modifications, GE of FcγRIIIa offers a host-directed strategy that could potentially enhance the efficacy of any FcγRIIIa-dependent IgG1 therapy. This generalizability, combined with swainsonine’s oral bioavailability and established safety profile, reinforces host-directed GE as a clinically tractable approach.^42^ More broadly, these findings point to an important role for the biomechanics of FcγR-dependent immune functions. As the field advances, integrating mechanobiological metrics into antibody development pipelines may enable mAb therapies that function more effectively within the dynamic mechanical environment of human physiology.

### Limitations of the study

The correlation between force durability and therapeutic efficacy, while compelling, does not preclude contributions from other effects of GE, particularly the role of interference with immunosuppressive lectin signaling. The degree to which preventing expression of sialylated N-glycans, specifically, enhanced mAb activity was not a focus of this study but likely contributed to the potentiation of therapeutic activity by swainsonine. There is also much left to learn about the determinants of force resistance in IgG-FcγR interactions. Future studies should incorporate biomechanical models to investigate how force durability influences immune cell trafficking, synapse formation, and target depletion. Additionally, the effect of GE on specific immune cell populations was not the focus of this study and warrants further evaluation. For example, determining how GE impacts macrophage, dendritic cell, or CD8 T cell function (all of which can express FcγRIIIa) will be important in future work.

## RESOURCE AVAILABILITY

### Lead contact

Requests for further information and resources should be directed to and will be fulfilled by the lead contact, Taia T. Wang (taiawang@stanford.edu).

### Materials availability

This study did not generate any new, unique reagents.

## STAR★METHODS

### EXPERIMENTAL MODEL AND STUDY PARTICIPANT DETAILS

#### Mouse model

All *in vivo* experiments were performed in 8- to 10- week-old, sex-matched, FcγR-humanized C57BL/6 mice that express human CD20 (hFcγR/hCD20) or hFcγR/hCD20 without Fc*γ*RIIIa (hFcγR/hCD20/FcγRIIIa^KO^). Mice were housed at 72°F and ambient humidity in a 12-hours light/dark cycle with free access to food and water. Both sexes of mice were used throughout the study. No significant differences were noted between sexes. All *in vivo* experiments were performed in compliance with the federal laws and institutional guidelines and have been approved by the Stanford University Institutional Animal Care and Use Committee (IACUC), specifically known as Administrative Panel on Laboratory Animal Care (APLAC).

#### Generation of hFcγR/hCD20/hFcγRIIIa^KO^ line

To generate the hFcγR/hCD20/FcγRIIIa^KO^ line, we crossed the existing hFcγR/hCD20 mice with the hFcγR/FcγRIIIa^KO^ mice, creating pups that are heterozygous for FcγRIIIa^KO^ while expressing the rest of hFcγR in addition to hCD20. These pups were then intercrossed to obtain pups homozygous for the FcγRIIIa knock-out. The resulting genotype is: hCD20+; hFcγRI+; hFcγRIIa+; hFcγRIIIa^KO^; hFcγRIIb+; hFcγRIIIb+; murine FcγR loci KO.

#### Human PBMCs

PBMCs from healthy adult donors were isolated from buffy coats obtained through the Stanford Blood Center. All samples were deidentified and collected under Stanford Blood Center’s institutional protocols with informed consent. Due to the anonymous nature of the buffy coat, detailed demographic information was not collected for these samples. Buffy coats were processed using SepMate™-50 according to manufacturer’s protocol to obtain fresh PBMCs.

### METHOD DETAILS

#### Antibodies and recombinant FcγRIIIa production

α-CD20, α-CD4, and α-CD25 hIgG1 mAbs, as well as recombinant FcγRIIIa were produced using the Expi293 Expression System. Plasmids encoding the rituximab clone were used for the α-CD20 hIgG1 mAbs, plasmids encoding the YTS191 clone was used for the α-CD4 hIgG1 mAbs. Plasmids encoding the PC-61 clone with G236A mutation was used to make the α-CD25 hIgG1 mAbs. To make the afucosylated version of the antibodies, a fucosyltransferase inhibitor was added into the culture media. IgG was purified using Protein G Sepharose, eluted with pH 2.7 buffer, and neutralized with Tris-HCl, pH 9. Recombinant FcγRIIIa was produced from lab-made pSC-sfGFP-oriFCGR3A plasmids, with glycosylation modified using 10μg/ml swainsonine. FcγRIIIa was purified with Ni Sepharose beads and eluted with 500 mM imidazole. Ultra centrifugal filters were used for buffer exchange and protein concentration.

#### ADCC assay

Fresh PBMCs were pre-treated with 10μg/ml of Swainsonine for 24 hours with 2ng/ml IL-2 supplement. EL4-hCD20 cells were stained with CFSE dye and opsonized with 2μg/ml of α-CD20 IgG, afucosylated α-CD20 IgG or trastuzumab in RPMI 1640 medium supplemented with 10% ultra-low IgG FBS and 1% penicillin-streptomycin for 1 hour, followed by PBS rinsing to remove unbound antibodies. Pre-treated PBMCs and opsonized EL4-hCD20 cells were co-cultured in a 10:1 ratio for 24 hours. ADCC was assessed by staining cells with LIVE/DEAD Fixable Near-IR dye and analyzing CFSE and Near-IR double-positive cells by flow-cytometry to measure specific cancer cell death.

#### ADCC assay with FcγR blocking

Prior to co-culture of PBMCs and EL4-hCD20 cells, PBMCs were coated with 10μg/ml of α-FcγRIIIa (clone 3G8), α-FcγRII (clone AT10), α-FcγRI (clone 10.1) or isotype control for 1 hour. The subsequent procedure followed the ADCC assay protocol.

#### NK cell degranulation assay

Fresh PBMCs were pre-treated with 10mg/ml Swainsonine for 24 hours with 2ng/ml IL-2 supplement. EL4-hCD20 cells were opsonized with 2mg/ml of α-CD20 or afucosylated α-CD20 in complete medium supplemented with ultra-low IgG fetal bovine serum for 1 hour, cells were rinsed with PBS afterwards to remove unbound antibodies. Swainsonine pre-treated PBMCs and opsonized EL4-hCD20 were co-cultured in a 10:1 ratio, with 2ml of CD107a antibody added. After 1 hour of co-culture, GolgiSTOP was added to the culture media and the cells were allowed to co-incubated for further 4 to 6 hours. Afterwards, cells were prepared and stained with α-CD56, α-CD19, α-CD3, α-CD14 and LIVE/DEAD Fixable Violet dye for flow cytometry analysis. Median fluorescence intensity (MFI) of CD107a expression on NK cells (CD56^+^CD3^−^CD14^−^CD19^−^) was measured.

#### Endogenous B or T cell depletion

hFcγR/hCD20 mice received daily swainsonine (1mg/kg) via oral gavage. α-CD20 (6mg/kg) or α-CD4 (clone YTS191, 2ug per mouse) was given on day 4. For B cell analysis, mice were sacrificed one week post α-CD20 treatment, and blood, spleen and inguinal lymph nodes were collected. Spleen and inguinal lymph nodes were homogenized into single cells. For T cell analysis, mice were sacrificed five days following α-CD4 administration. Cells from blood and spleen underwent ACK lysis to remove red blood cells. The cells were stained with APC α-human CD20, FITC α-mouse CD3, PerCP/Cyanine5.5 α-mouse NK1.1, Alexa Fluor 700 α-mouse CD45 and DAPI, followed by flow cytometry analysis.

#### α-CD25 mediated Treg depletion

Fc*γ*R-humanized mice were subcutaneously implanted with 2 x 10^5^ B16F10 cells. Mice received daily swainsonine (1mg/kg) via oral gavage from day 10, and two doses of 100 μg of α-CD25 (afucosylated, G236A) on day 12, 14 via intraperitoneal injection. Tumors were harvested on day 15 and dissociated for assessing the frequency of Tregs (CD4^+^Foxp3^+^).

#### Mannose recognition via lectin flow cytometry

Recombinant His-tagged mouse mannan-binding lectin (MBL-2) and recombinant mouse macrophage mannose receptor (MMR/CD206) were used to assess binding to high-mannose glycans as a surrogate for potential complement-dependent and phagocyte-mediated clearance pathways, respectively. EL4-hCD20 cells were treated with swainsonine (10 μg/mL) for 24 hours, washed, and incubated with MBL-2 (10 μg/mL) or CD206 (5 μg/mL) for 30 min at 37 °C. Bound lectins were detected using FITC-conjugated α-His antibody, followed by flow cytometric analysis. All staining steps were performed in PBS supplemented with Ca^2+^ and Mg^2+^.

#### PBMC characterization post-SW treatment

PBMCs were treated with 10 μg/ml of Swainsonine for 24 hours, followed by flow cytometry analysis of immune cells composition using BUV615 α-human CD16, PE α-human CD32, PE-Cy7 α-human CD32b,c, FITC α-human CD64, BV650 α-human CD11b, BV605 α-human CD14, BV711 α-human CD56, AF700 α-human CD19, BUV737 α-human CD11c, LIVE/DEAD Fixable Blue Dead Cell Stain Kit, BV480 α-human MHC Class II (HLA-DR), BUV395 α-human CD33, BV421 α-human CD8, BV570 α-human CD4.

#### Immune cell characterization post-SW treatment in mice

hFcγR/hCD20 mice 8-10 weeks old received daily swainsonine (1mg/kg) via oral gavage for a week, then were sacrificed to collect whole blood and spleen. Spleen was homogenized into singe cells. Both blood cells and homogenized spleen cells underwent ACK lysis to remove red blood cells. The cells were stained with BUV496 α-mouse CD3, BV650 α-mouse CD4, BUV737 α-mouse CD8b, PE-Cy5 α-mouse NK1.1, Pacific Blue α-mouse CD19, BUV563 α-mouse CD11b, PE-Cy7 α-mouse Ly6C, APC/Cy7 α-mouse Ly6G, BUV661 α-mouse, BUV395 α-mouse I-A/I-E, BV785 α-mouse F4/80, BV711 α-mouse CD86, BV605 α-human CD16, PE α-human CD32, FITC α-human CD64, APC α-mouse CD107a, AF700 α-human CD20 and LIVE/DEAD Fixable Aqua Dead Cell Stain, followed by flow cytometry analysis.

#### Endogenous antibody measurement in cancer-bearing mice

hFcγR/hCD20 mice 8-10 weeks old with subcutaneously implanted EL4-hCD20 were bled on day 10 via retro-orbital bleeding into Lithium Heparin-treated tubes. Serum was collected after centrifugation at 1500 x g for serum collection. EL4-hCD20, Hepa 1-6, MC38 and B16F10 cells were incubated with the mouse serum, stained with secondary FITC α-mouse IgG, and analyzed for endogenous antibody binding via flow cytometry.

#### Cell viability assay (CellTiter-Glo assay)

Fresh PBMCs were treated with increasing concentrations of swainsonine for 24 hours, followed by cell viability analysis using the CellTiter-Glo assay as per manufacturer’s instruction.

#### Lectin flow cytometry

Hippeastrum hybrid (Amaryllis) (HHA) was either labeled with Pacific Orange or biotinylated as per manufacturer’s instruction and was used for detection of high mannose glycans. Biotinylated Erythrina Cristagalli Lectin (ECL, ECA) was used for galactose detection. Cells were stained with Pacific Orange-labeled or biotinylated lectins followed by streptavidin-APC as secondary staining if biotinylated HHA was used. The expression of high mannose glycans on the cell surface was determined by the median fluorescence intensity of the HHA signal as determined by flow cytometry.

#### Mass spectrometric analysis of mAb glycosylation

For quantitative analysis of the glycoforms at the N297 site of IgG1, multiple-reaction monitoring (MRM) analysis for selected target glycopeptides and their glycoforms was applied using the nanoLC-4000 QTRAP platform to the samples, which had been digested with trypsin. The mass/charge ratio (m/z) of four-charged ions for all different glycoforms as Q1 and the fragment ion at m/z 366.1 asQ3 for each of transition pairs were used for the MRM assays. A native IgG tryptic peptide (131-GTLVTVSSASTK-142) with Q1/ Q3 transition pair of 575.9+2/780.4 was used as a reference peptide for normalization. All raw MRM data were processed using MultiQuant 2.1.1 (SCIEX). All MRM peak areas were automatically integrated and inspected manually. In the case where the automatic peak integration by MultiQuant failed, manual integration was performed using the MultiQuant Software.

#### Mass spectrometric analysis for recombinant FcγRIIIa glycosylation

FcγRIIIa N-glycan (as a whole protein) analysis was performed by releasing glycans via PNGaseF digestion after reduction, alkylation, and solid-phase extraction. Samples (5 μg each) were dried, reduced with SDS and DTT, and alkylated with iodoacetamide in the dark. The reactions were quenched, acidified, and incubated at −20°C before being processed using S-trap SPE columns. Released glycans were eluted, dried, reconstituted in water, and labeled with procainamide using the LudgerTag PROC Glycan Labeling Kit. Labeled glycans were diluted and analyzed using HILIC-LC/MS/MS. Separation was achieved on an XBridge Premier BEH Amide VanGuard column with a gradient of 76%-51% acetonitrile at 400 μL/min. An Orbitrap Q-Exactive HF mass spectrometer operated in positive ion mode with data-dependent acquisition (DDA) was used to analyze the samples. The MS scan range was m/z 500–3500 with 60k resolution, and the “Top 10” ions were selected for HCD fragmentation. Data acquisition was managed using Xcalibur 4.5 software. Glycan identification involved SimGlycan software, searching against the antibody database with parameters including a 10 ppm mass tolerance, [M+H]+ adducts, and a procainamide delta mass of 219.173 Da. Only glycans with a Proximity Score ≥ 70 were considered. Identified glycoforms were manually validated, and their relative abundances were quantified based on peak area integration. This workflow provided accurate glycoform characterization and distribution insights.

FcγRIIIa N-glycan (specifically at position N45 and N162) analysis was performed first by in-solution digestion conducted using Glu-C and chymotrypsin enzymes. For Glu-C digestion, protein aliquots were reduced with DTT in urea and sodium phosphate, alkylated with iodoacetamide, and quenched with DTT. Urea was diluted, and Glu-C was added for overnight digestion at 33°C. For chymotrypsin digestion, samples were reduced and denatured in guanidine hydrochloride, alkylated, and quenched similarly. Guanidine concentration was reduced, and chymotrypsin was added for a 2-hour digestion at 25°C. Both digests were acidified with formic acid and cleaned via solid-phase extraction using SOLA HRP cartridges. Proteins were identified by nano LC/MS/MS using an Orbitrap Fusion mass spectrometer coupled with a Dionex RSLCnano system. Peptides were separated on a C-18 column, eluted with a gradient of acetonitrile and formic acid, and analyzed using a data-dependent acquisition (DDA) method. The Orbitrap operated with high resolution for precursor selection and fragment ion analysis, employing HCD and EThcD fragmentation methods. Data were analyzed using Byonic software with the Homo sapiens Uniprot database and glycan searches against 132 N-linked glycans. Parameters included missed cleavages, carbamidomethylation, and variable modifications. Peptides were filtered to a 1% FDR, and glycoforms were manually validated. Quantification was based on peak area integration from extracted ion chromatograms.

#### Antibody-binding to FcγRIIIa^+^ or FcγRIIIa^−^ Jurkat cells assessed by flow-cytometry

α-CD20 or afucosylated α-CD20 were conjugated using a FITC conjugation kit. Jurkat WT or FcγRIIIa-expressing Jurkat cells were treated with 0, 2, 10, 50mg/ml Swainsonine for 24 hours, then stained with FITC-conjugated α-CD20 or afucosylated α-CD20, followed by flow cytometry analysis of antibody binding.

#### Immunohistochemical (IHC) analysis

Prior to paraffin embedding, tissues were fixed in 4% paraformaldehyde and washed with 75% ethanol. Heated paraffin sections were dewaxed in xylene followed by ethanol gradation. Antigens were retrieved in 1mM EDTA buffer (pH 7.8) by boiling for 15 minutes. Following blocking endogenous enzymes with 3% hydrogen peroxide and preventing non-specific binding with 2% bovine serum albumin (BSA), slides were stained with α-NK1.1 overnight at 4°C. For horseradish peroxidase (HRP) immunodetection, slides were stained with EnVision+ System- HRP Labelled Polymer and developed with 3,3’-Diaminobenzidine tetrahydrochloride. Slides were counterstained with hematoxylin and were imaged on a Keyence BZ-X810 microscope.

#### Lectin-ELISA

Cells were treated with 10mg/ml of Swainsonine for 24 hours, after which cell lysate were prepared with NP40 lysis buffer according to manufacturer’s protocol. The protein concentration of the cell lysate was determined using a Bradford assay. Cell lysates were then diluted to 2mg/ml with PBST (PBS with 0.1% Tween20) /0.1% Brij35 supplemented with Halt^™^ protease inhibitor. To perform the Lectin-ELISA, half-volume 96 well-plates were coated with 2mg/ml α-FcγRIIIa (clone 3G8) in PBS overnight at 4°C. The plates were then blocked with a Protein-Free Blocking Buffer for 1 hour at room temperature. Cell lysates were then added to 3G8-coated wells and incubated overnight at 4°C on shaking platform. Following incubation, the wells were rinsed with PBST, and 20mg/ml biotinylated HHA and biotinylated α-FcγRIIIa (clone GRM1) in PBST/0.1% BSA were added to their respective wells and allowed to bind for 2 hours at room temperature on a shaking platform. After rinsing with PBST, Streptavidin-AP was added and allowed to bind for 1 hour at room temperature. Lastly, after rinsing with PBST, substrate prepared from diluting PNPP Substrates in Diethanolamine Substrate Buffer was added to reveal binding signal. Measurement was taken with a plate reader at wavelength 405nm. The signal intensity represents HHA binding and FcγRIIIa protein expression on the captured FcγRIIIa from the protein lysate respectively.

#### Solid tumor model with intertumoral swainsonine administration

5 × 10^5^ EL4-hCD20 cells were resuspended in a 1:1 Matrigel Matrix and PBS solution and implanted subcutaneously into the flanks of hCD20/FcγR-humanized mice. Tumors were allowed to establish for 8 days then distributed into the different groups. Swainsonine was given intratumorally at 2mg/kg in PBS thrice per week. α-CD20 hIgG1 or afucosylated α-CD20 hIgG1 were given once on day 10 at 6mg/kg. The tumor size was monitored on every other day and volume was calculated as 0.5 x length x width^2^ using caliper measurements. Mice were sacrificed at the end of experiment and the tumors were excised for photo record and were then placed into 4% paraformaldehyde for fixation, followed by storage in 75% ethanol.

#### Hematologic tumor model

5 x 10^5^ EL4-hCD20-Luc cells with luciferase reporter were resuspended in 200ul of PBS and intravenously implanted into hFcγR/hCD20 mice or hFcγR/hCD20/FcγRIIIa^KO^ mice by tail-vein injection. Swainsonine was given daily via oral gavage at 1mg/kg thrice per week. Afucosylated α-CD20 was given on day 6 post implantation at 6mg/kg via intraperitoneal injection. The mice were monitored daily for survival analysis.

#### Bioluminescence imaging

Mice with EL4-hCD20-Luc cells implanted were injected with 150mg/kg D-Luciferin, Potassium Salt in PBS and the bioluminescence signals from the implanted cancer cells was visualized using the Lago X In Vivo Imager.

#### NK cell depletion in intravenous tumor model

Isotype control or an NK cell depletion antibody (α-NK1.1) was administered intraperitoneally starting 4 days prior to the intravenous implantation of 5 x 10^5^ EL4-hCD20-Luc cells. The initial dose was 200μg, followed by 100μg administered 1 day prior to the implantation of EL4-hCD20-Luc cells. After that, mice were given 100μg twice per week. Afucosylated α-CD20 was given on day 6 post implantation at 6mg/kg via intraperitoneal injection. To confirm NK cell depletion, blood was drawn from mice on day 7 to assess for NK cell percentage (CD49b+).

#### Microfluidics chamber

Microfluidics chamber assay was performed similarly to prior work.^25^ Briefly, 35mm tissue culture dishes were coated overnight at 4° C with 10ug/mL of either α-CD20 or afucosylated α-CD20 IgG in PBS, or with PBS alone as negative control. Plates were then washed twice with PBS, followed by blocking in 5% milk in PBST overnight at 4° C. On the day of experiment, these plates were rinsed twice with PBS and kept moist with enough PBS to cover the surface. A parallel plate flow chamber with gasket B (flow width: 0.25cm, gasket thickness: 0.0254cm) was mounted onto the IgG coated or uncoated 35mm tissue culture dishes. For the assay, Jurkat FcgRIIIa^−^ or Jurkat FcgRIIIa^+^ cells pre-treated in separate flasks with DMSO (control) or 10 ug/mL swainsonine, followed by rinsing in PBS and resuspended in 2 x 10^5^/ml in base media (IMDM) were used. The cells were pushed into the tubing upstream of the flow chamber via a syringe. PBS was then introduced into the chamber at a controlled flow rate using a syringe pump AL-1000 fitted with a 50ml syringe, initially 200ul at 1ml/min to move cells into the chamber. Cells were then allowed to bind to the antibody on the coated surface undisturbed for 5 mins. After that, PBS was pumped through the chamber at increasing flow rate, maintaining each flow rate for 30 seconds before increasing stepwise by a factor of 2.152 to create wall shear stresses from 0.2 to 115.11 dynes/cm^2^, or until all cells were detached. Data was collected on the same days repeated 3-4 times for each condition being compared. Videos of cell binding were recorded at 2 frames per second, and cells were manually quantified in one frame. Cell counts were taken immediately after the initial 5 mins settling time was defined as the initial number of cells that arrived at the chamber. Cell counts taken at 15-20 seconds after applying a 0.2 dynes/cm^2^ shear stress to gently wash away unbound cells was defined as the 100% bound cells for subsequent measurement. Once the flow rate increment has begun, cell count was taken at the end of each 30-seconds interval, and these cell counts were normalized to the cell number defined as the 100% bound cells to calculate the percentage of cells remaining bound at each flow rate.

### QUANTIFICATION AND STATISTICAL ANALYSIS

All statistical analyses were conducted using Prism 9.01 (GraphPad). Sample sizes and statistical tests are indicated in the figure legends. Data were considered statistically significant at P<0.05.

## Supplementary Material

1

Supplemental information can be found online at https://doi.org/10.1016/j.immuni.2026.03.028.

## Figures and Tables

**Table:** KEY RESOURCES TABLE

REAGENT or RESOURCE	SOURCE	IDENTIFIER
Antibodies		
Human IgG1 monoclonal anti-CD20	Produced in house	N/A
Human IgG1 monoclonal anti-CD4	Produced in house	N/A
Human IgG1 monoclonal anti-CD25	Produced in house	N/A
Trastuzumab	Selleck Chemicals	Cat# A2007
Mouse anti-human FcγRIIIa clone 3G8	Fisher Scientific	Cat#NC1538761
Mouse anti-human FcγRII clone AT10	Bio-Rad Laboratories	Cat# MCA1075
Mouse anti-human FcγRI clone 10.1	Thermo Fisher Scientific	Cat#16-0649-85
Mouse IgG1 isotype control	Thermo Fisher Scientific	Cat#16-4714-85
BV785 anti-human CD107a	BioLegend	Cat#328644; RRID:AB_2565968
PE anti-human CD56 (NCAM)	BioLegend	Cat#362524; RRID:AB_2564161
Alexa Fluor 700 anti-human CD19	BioLegend	Cat#363034; RRID:AB_2616936
Alexa Fluor 700 anti-human CD3	BioLegend	Cat#317340; RRID:AB_2563408
Alexa Fluor 700 anti-human CD14	BioLegend	Cat#367114; RRID:AB_2566716
APC anti-human CD20	BioLegend	Cat#302310; RRID:AB_314258
FITC anti-mouse CD3	BioLegend	Cat#100204; RRID:AB_312661
PerCP/Cyanine5.5 anti-mouse NK1.1	BioLegend	Cat#156526; RRID:AB_2894655
Alexa Fluor 700 anti-mouse CD45	BioLegend	Cat#103128; RRID:AB_493715
FITC anti-His	BioLegend	Cat#362618; RRID:AB_2904384
BUV615 anti-human CD16	BD Biosciences	Cat#751572; RRID:AB_2875567
PE anti-human CD32	BioLegend	Cat#303205; RRID:AB_314337
PE-Cy7 anti-human CD32b,c	BioLegend	Cat#398314; RRID:AB_2890844
FITC anti-human CD64	BioLegend	Cat#305006; RRID:AB_314490
BV650 anti-human CD11b	BioLegend	Cat#101259; RRID:AB_2566568
BV605 anti-human CD14	BioLegend	Cat#367126; RRID:AB_2716231
BV711 anti-human CD56	BioLegend	Cat#318336; RRID:AB_2562417
BUV737 anti-human CD11c	BD Biosciences	Cat#568328; RRID:AB_3684184
BV480 anti-human MHC Class II (HLA-DR)	BD Biosciences	Cat#746360; RRID:AB_2743679
BUV395 anti-human CD33	BD Biosciences	Cat#568374; RRID:AB_3684223
BV421 anti-human CD8	BioLegend	Cat#344747; RRID:AB_2629583
BV570 anti-human CD4	BioLegend	Cat#300534; RRID:AB_2563791
BUV496 anti-mouse CD3	BD Biosciences	Cat#741117; RRID:AB_2870707
BV650 anti-mouse CD4	BD Biosciences	Cat#563747; RRID:AB_2716859
BUV737 anti-mouse CD8b	BD Biosciences	Cat#741811; RRID:AB_2871149
PE-Cyanine5 anti-mouse NK1.1	BioLegend	Cat#108716; RRID:AB_493590
Pacific Blue anti-mouse CD19	BioLegend	Cat#152416; RRID:AB_2927869
BUV563 anti-mouse CD11b	BD Biosciences	Cat#741242; RRID:AB_2870793
PE-Cyanine7 anti-mouse Ly6C	BioLegend	Cat#128018; RRID:AB_1732082
APC/Cyanine7 anti-mouse Ly6G	BioLegend	Cat#127624; RRID:AB_10640819
BUV661 anti-mouse CD11c	BD Biosciences	Cat#750449; RRID:AB_2874610
BUV395 anti-mouse I-A/I-E	BD Biosciences	Cat#743876; RRID:AB_2741827
BV785 anti-mouse F4/80	BioLegend	Cat#123141; RRID:AB_2563667
BV711 anti-mouse CD86	BD Biosciences	Cat#740688; RRID:AB_2734766
BV605 anti-mouse CD16	BioLegend	Cat#302040; RRID:AB_2562990
APC anti-mouse CD107a	BioLegend	Cat#121614; RRID:AB_2234505
Alexa Fluor 700 anti-human CD20	BioLegend	Cat#302322; RRID:AB_493753
FITC anti-mouse IgG	BioLegend	Cat#406001; RRID:AB_315029
Anti-mouse NK1.1	Cell Signaling	Cat#24395
Anti-human FcyRIIIa (clone 3G8)	Invitrogen	Cat#16-0166-82; RRID:AB_2573072
Anti-human FcyRIIIa (clone GRM1)	SouthernBiotech	Cat#9570-08
Mouse IgG2a isotype control	Bioxcell	Cat#BE0085
Mouse IgG2a anti-NK1.1	Bioxcell	Cat#BE0036
FITC Anti- CD49b	BD Biosciences	Cat#553857; RRID:AB_395093
Bacterial and virus strains		
N/A	N/A	N/A
Biological samples		
Healthy human peripheral blood mononuclear cells	Stanford Blood Center	N/A
Chemicals, peptides, and recombinant proteins		
Fucosyltransferase inhibitor	Millipore Sigma	Cat#344827-10MG
Protein G Sepharose	Cytiva	Cat# GE17-0618-02
pH 2.7 buffer	Teknova	Cat#P3727
Tris-HCl, pH9	Teknova	Cat#T1090
Swainsonine	Cayman Chemical	Cat#16860
Ni Sepharose beads	Milipore Sigma	Cat#GE1-5268-01
Ultra centrifugal filters	Milipore Sigma	Cat#UFC903008/UFC901008
IL-2	Miltenyi Biotec	Cat#130-097-742
CFSE dye	Thermo Fisher Scientific	Cat#C34554
LIVE/DEAD Fixable Near-IR dye	Thermo Fisher Scientific	Cat#L34975
GolgiSTOP	BD Biosciences	Cat#554724
LIVE/DEAD Fixable Violet dye	Thermo Fisher Scientific	Cat#L34955
ACK Lysing Buffer	Thermo Fisher Scientific	Cat#A1049201
DAPI	Invitrogen	Cat#D21490
Recombinant His-tagged mouse mannan-binding lectin (MBL-2)	SinoBiological	Cat#50063-M07H
Recombinant His-tagged mouse macrophage mannose receptor (MMR/CD206	R&D Systems	Cat#2535-MM-050/CF
LIVE/DEAD Fixable Blue Dead Cell Stain Kit	Invitrogen	Cat#L23105
LIVE/DEAD Fixable Aqua Dead Cell Stain	Invitrogen	Cat#L34957
Hippeastrum hybrid (Amaryllis) (HHA) lectin	Vector Laboratories	Cat# L-1380
Pacific Orange labeling dye	Thermo Fisher Scientific	Cat#P30014
Biotin protein labeling kit	Roche	Cat#11418165001
Erythrina Cristagalli lectin (ECL, ECA), biotinylated	Vector Laboratories	Cat# B-1145
Streptavidin-APC	BioLegend	Cat#405207
FITC conjugation kit	Abcam	Cat#ab188285
EnVision+ System- HRP Labelled Polymer	Agilent	Cat#K4003
NP40 lysis buffer	Thermo Fisher Scientific	Cat# J60766.AP
Bradford Assay Kit	Thermo Fisher Scientific	Cat#1863028
Brij35	Thermo Fisher Scientific	Cat#85117
Halt^™^ protease inhibitor	Thermo Fisher Scientific	Cat#78430
Protein-Free Blocking Buffer	Thermo Fisher Scientific	Cat#37584
Streptavidin-AP	SouthernBiotech	Cat#7105-04
PNPP Substrates	Thermo Scientific	Cat#34047
Diethanolamine Substrate Buffer	Pierce	Cat#34064
Matrigel Matrix	Corning	Cat#356230
LudgerTag PROC Glycan Labeling Kit	Ludger Ltd.	Cat#LT-KPROC-24
D-Luciferin, Potassium Salt	Goldbio	Cat# LUCK-1G
Critical commercial assays		
CellTiter-Glo assay	Promega	Cat#G7571
Experimental models: Cell lines		
Human: Expi293 Expression System	ThermoFisher Scientific	Cat#A14635
Mouse: EL4-hCD20 mouse lymphoma, overexpressing human CD20	Modified in House	N/A
Mouse: B16F10 mouse melanoma	ATCC	Cat#CRL-6475
Mouse: Hepa 1-6 mouse hepatoma	ATCC	Cat#CRL-1830
Mouse: MC38 murine colon adenocarcinoma	Millipore Sigma	Cat#SCC172
Experimental models: Organisms/strains		
Mouse: FcγR-humanized C57BL/6 that express human CD20 (hFcγR/hCD20)	In-house	N/A
Mouse: hFcγR/hCD20 without Fc*γ*RIIIa (hFcγR/hCD20/FcγRIIIa^KO^)	In-house	N/A
Oligonucleotides		
N/A	N/A	N/A
Recombinant DNA		
Plasmid: anti-CD20 hIgG1 monoclonal (clone rituximab)	Dr. J. Ravetch	N/A
Plasmid: anti-CD4 hIgG1 monoclonal (clone YTS191)	Dr. S. Bournazos	N/A
Plasmid: anti-CD25 hIgG1 monoclonal (clone PC-61, with G236A mutation)	Dr. R. Dehan	N/A
Plasmid: human recombinant FcγRIIIa	Produced in-house	pSC-sfGFP-oriFCGR3A
Software and algorithms		
FlowJo v 10.9.0	Tree Star	https://www.flowjo.com/flowjo/download
Prism v10	GraphPad Software	http://www.graphpad.com/
MultiQuant 2.1.1	SCIEX	https://sciex.com/products/software/multiquant-software
Byonic software	Protein Metrics Inc.	RRID: SCR_016735
Xcalibur 4.5	Thermo Fisher Scientific	https://www.thermofisher.com/order/catalog/product/OPTON-30965
nanoLC-4000 QTRAP platform	SCIEX	https://sciex.com/products/mass-spectrometers/qtrap-systems/qtrap-4000-system
SimGlycan	PREMIER Biosoft	https://www.premierbiosoft.com/glycan/index.html
Other		
Lithium Heparin-treated tubes	BD Biosciences	Cat#365985
Plate flow chamber with gasket B (flow width: 0.25cm, gasket thickness: 0.0254cm)	Glycotech	Cat#1188-31-001
Syringe pump AL-1000	World Precision Instrument Aladdin	Cat#NC1881156
SepMate^™^-50	StemCell Technologies	Cat#3502254199

## Data Availability

• All data reported in this paper will be shared by the lead contact upon request. • This paper does not report any original code. • Any additional information required to reanalyze the data reported in this paper is available from the lead contact upon request.

## Highlights

- Preventing N-glycan maturation enhances antibody-mediated tumor cell depletion
- Glycoengineering potentiates efficacy of α-CD20 and α-CD25 in humanized cancer models
- Glycoengineering strengthens IgG-FcγRIIIa bond resilience under physiological force
- Force durability is a parameter for therapeutic antibodies beyond affinity optimization
