# Supplementary material for: Pharmacologic glycoengineering of Fcγ receptor IIIa enhances force-resistant IgG-FcγR interactions and anti-tumor antibody efficacy
Source: Immunity. Author manuscript; Available in PMC 2026 Jun 13. (PMC13261076; doi:10.1016/j.immuni.2026.03.028)
Supplement: 1 [file NIHMS2174710-supplement-1.pdf]

Immunity, Volume *59*

Supplemental information

Pharmacologic glycoengineering of Fcγ receptor IIIa enhances force-resistant IgG-FcγR interactions and anti-tumor antibody efficacy

Bowie Yik-Ling Cheng, Raquel M. Centeio, David Kung-Chun Chiu, Casey L. Kiyohara, Ella Herzog, Rony Dahan, Wendy E. Thomas, and Taia T. Wang

Figure S1

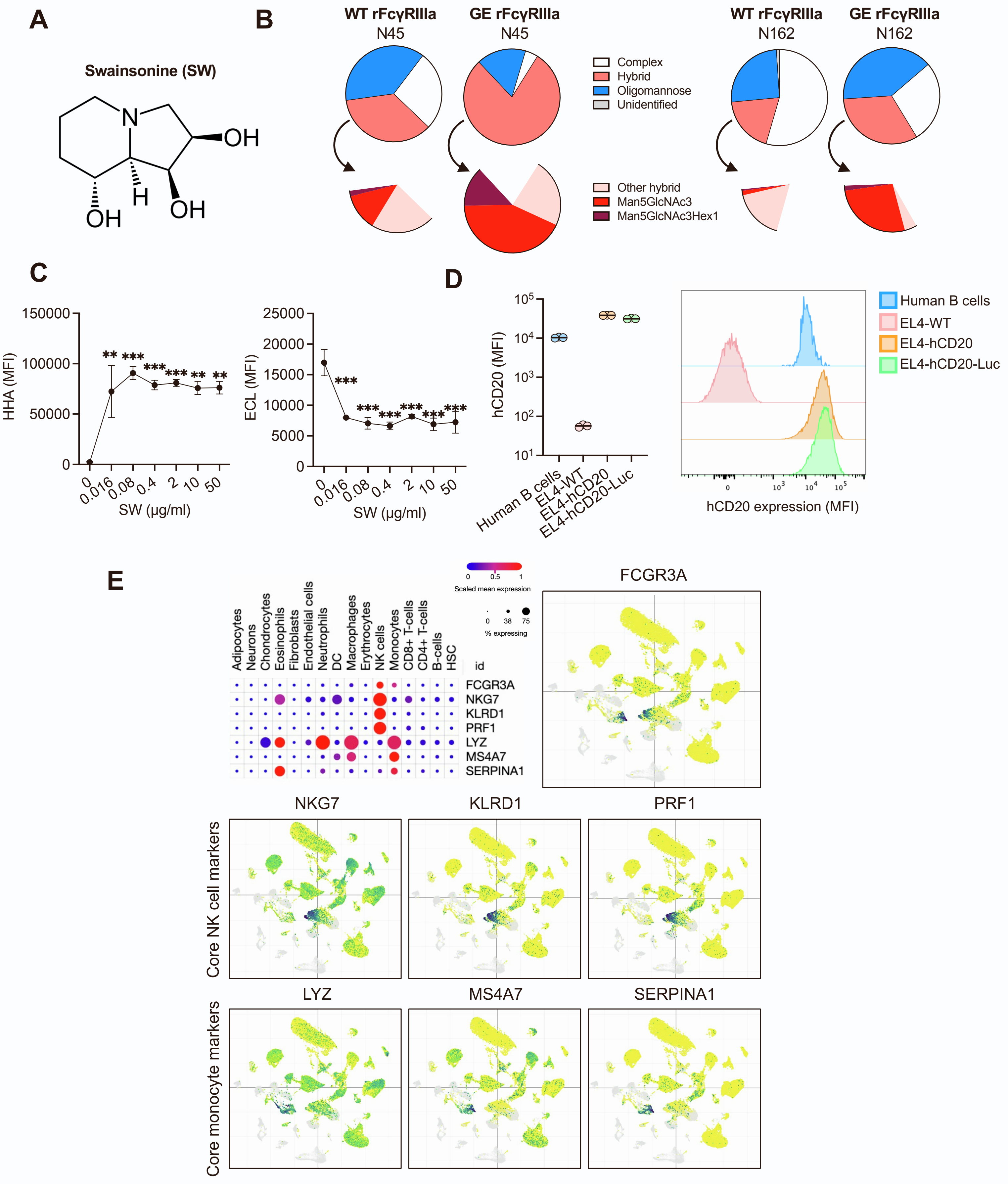
A
Swainsonine (SW)
B
WT rFcγRIIIa N45
GE rFcγRIIIa N45
WT rFcγRIIIa N162
GE rFcγRIIIa N162
Complex
Hybrid
Oligomannose
Unidentified
Other hybrid
Man5GlcNAc3
Man5GlcNAc3Hex1
C
HHA (MFI)
ECL (MFI)
SW (µg/ml)
D
hCD20 (MFI)
Human B cells
EL4-WT
EL4-hCD20
EL4-hCD20-Luc
hCD20 expression (MFI)
E
Scaled mean expression
% expressing
Adipocytes
Neurons
Chondrocytes
Eosinophils
Fibroblasts
Endothelial cells
Neutrophils
DC
Macrophages
Erythrocytes
NK cells
Monocytes
CD8+ T-cells
CD4+ T-cells
B-cells
HSC
id
FCGR3A
NKG7
KLRD1
PRF1
LYZ
MS4A7
SERPINA1
Core NK cell markers
Core monocyte markers

Figure S1: The effect of glycoengineering by swainsonine on FcγRIIIa glycosylation, whole cell glycosylation, and FCGR3A expression in human lymphoma samples, related to Figure 1 (A) Chemical structure of swainsonine (SW). (B) Proportion of different glycan types on position P45 (left) and N162 (right) of WT and SW-treated (Glycoengineered, GE) recombinant FcγRIIIa (rFcγRIIIa). (C) HHA (mannose-binding) and ECL (galactose-binding) lectin binding on SW treated human PBMCs. (D) Expression level of human CD20 on human B cells from three different donors, EL4-WT, EL4-hCD20 and EL4-hCD20-Luc cell lines. (E) Single-cell RNA-sequencing (scRNA-seq) data from B cell lymphoma patients obtained from the Broad Institute Single Cell Portal (SCP3385 [S1]). UMAP visualization shows immune cells derived from B cell lymphoma samples (highlighted in yellow) overlaid on a background of other tumor types (grey) included in the integrated dataset. Expression of FCGR3A is selectively enriched in NK cells and monocytes, with the highest expression observed in NK cells. Dot plot analysis (top left) summarizes scaled mean expression and the fraction of expressing cells for FCGR3A, canonical NK cell markers (NKG7, KLRD1, PRF1), and monocyte markers (LYZ, MS4A7, SERPINA1) across annotated immune populations. UMAP plots show the spatial distribution of FCGR3A and lineage-defining markers across the UMAP embedding, confirming that FCGR3A expression is largely restricted to NK cells and monocytes within the B cell lymphoma compartment. The data are represented as mean +/-SEM in (C). Statistical significance was calculated with one-way ANOVA with Dunnett's Post-Hoc test in (C). $*p < 0.05$, $**p < 0.01$, $***p < 0.001$.

Figure S2

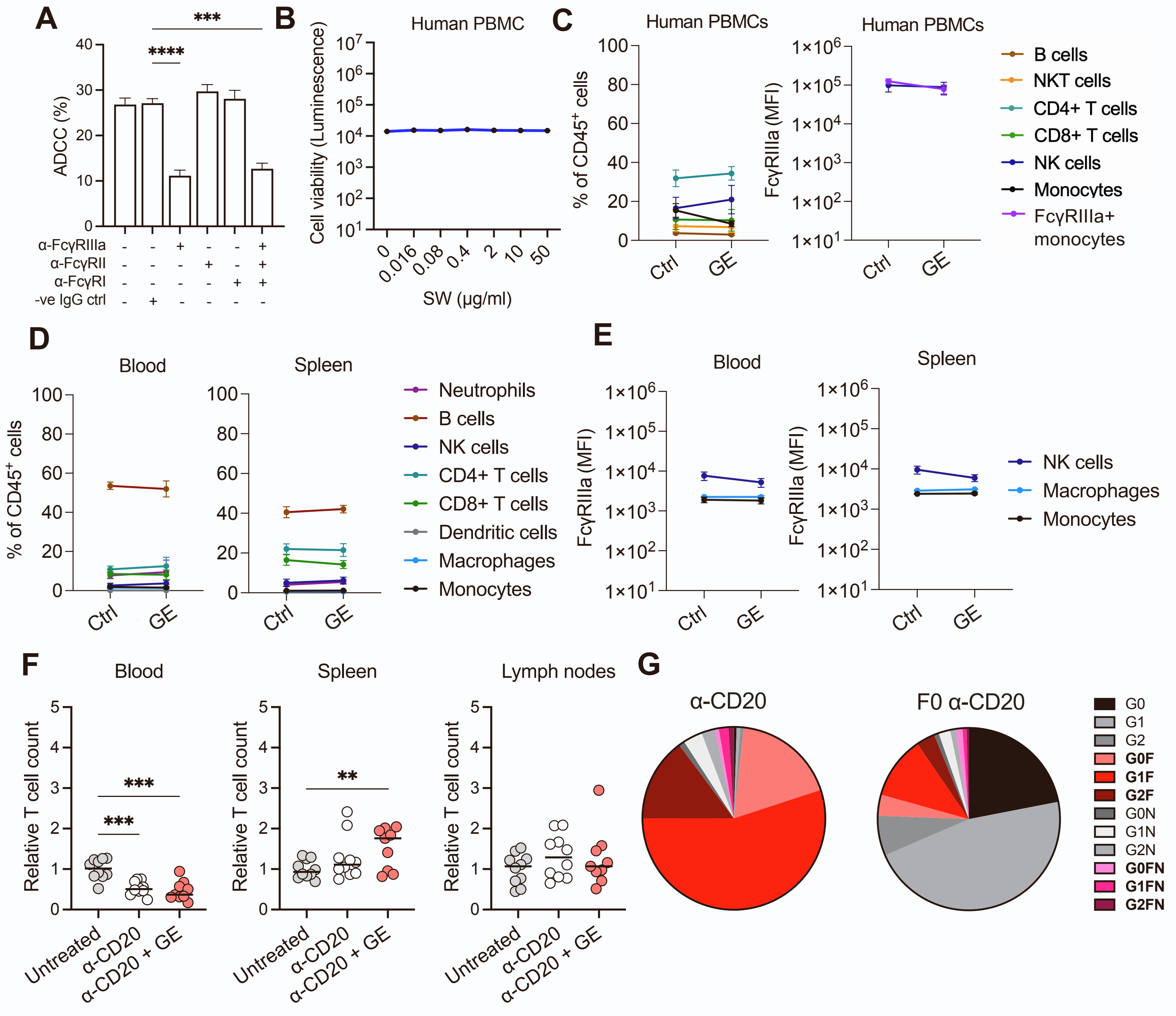

Figure S2: The effect of glycoengineering on immune cells and Fc glycan composition on recombinantly expressed mAbs, related to Figure 1 (A) ADCC response of PBMCs treated FcγR-blocking antibodies targeting FcγRIIIa, FcγRII or/and FcγRI, followed by co-culture with α-CD20-opsonized EL4-hCD20 cells. (B) CellTiter-Glo assay showing the viability of PBMCs treated for 24 hours with swainsonine (GE). (C) PBMCs treated with swainsonine for 24 hours followed by analysis on immune cell composition and FcγRIIIa expression on relevant cell types. (D) Immune cell composition and (E) FcγRIII expression on relevant cells in blood andspleen of hFcγR/hCD20 mice treated with daily oral swainsonine for one week. (F) T cells were quantified from blood, spleen and lymph nodes of hFcγR/hCD20 mice treated with α-CD20 alone or in combination with swainsonine (GE). (G) Fc glycan composition of (left) α-CD20 or (right) afucosylated (F0) α-CD20. IgG were made in house using a plasmid that encodes rituximab. F0 α-CD20 was made in the presence of fucosyltransferase inhibitor. (G: galactose, F: fucose, N: N-acetylglucosamine). The data are represented as mean +/-SEM in (A-E). Statistical significance was calculated with one-way ANOVA in (A, F), one-way ANOVA with Dunnett's Post-Hoc test in (B-E). **p* < 0.05, ***p* < 0.01, ****p* < 0.001.

Figure S3

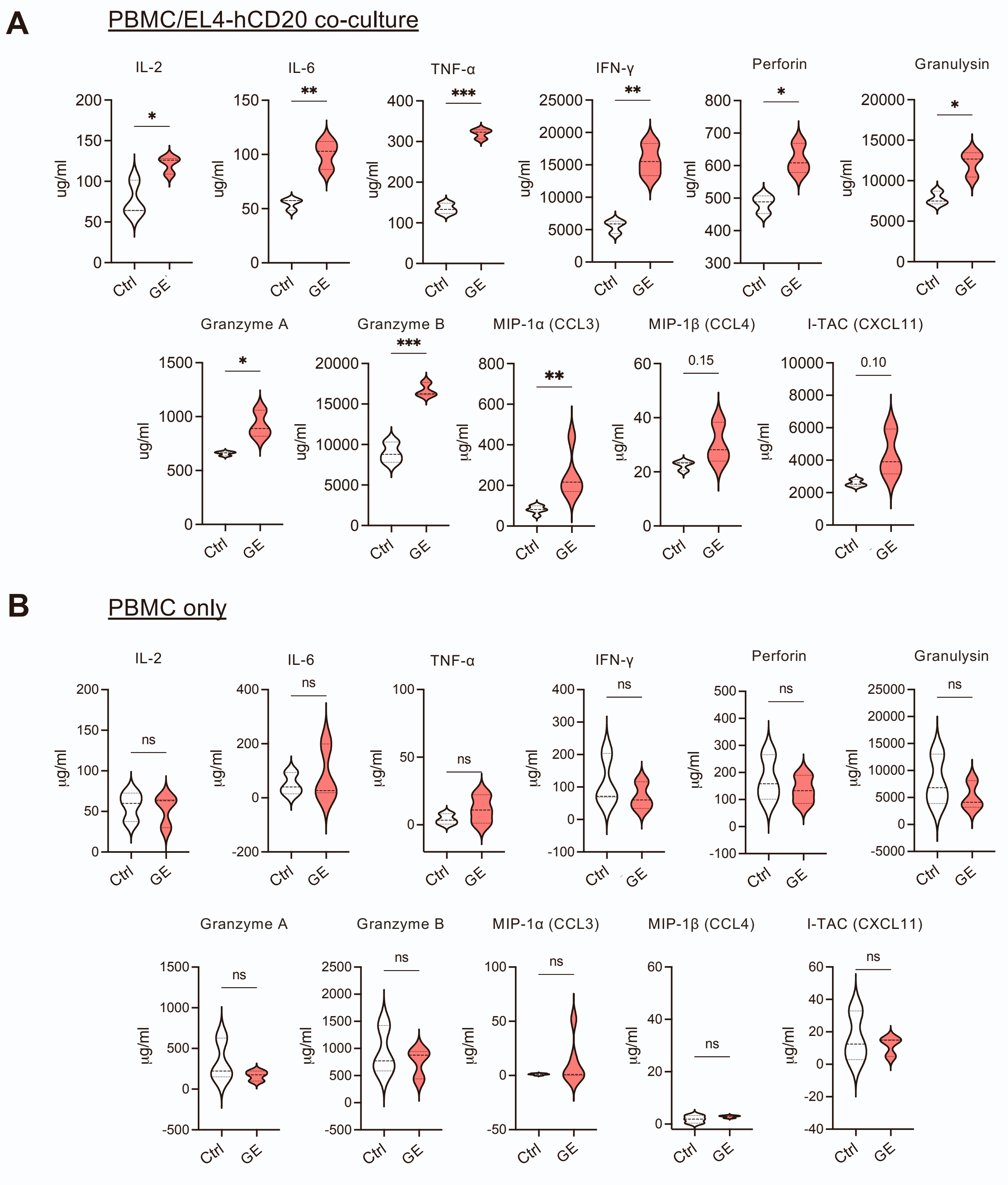

Figure S3: Effect of glycoenginnering on cytokine release from human PBMC, related to Figure 2 (A) Human cytokine released from the co-culture of swainsonine pre-treated (GE) or untreated human PBMCs with F0 α-CD20-opsonised EL4-hCD20 for 24 hours. (B) Human cytokine released from untreated or GE PBMCs. Statistical significance was calculated by two tailed unpaired student's T test in (A-B). *$p < 0.05$, **$p < 0.01$, ***$p < 0.001$, ns is non-significant.

Figure S4

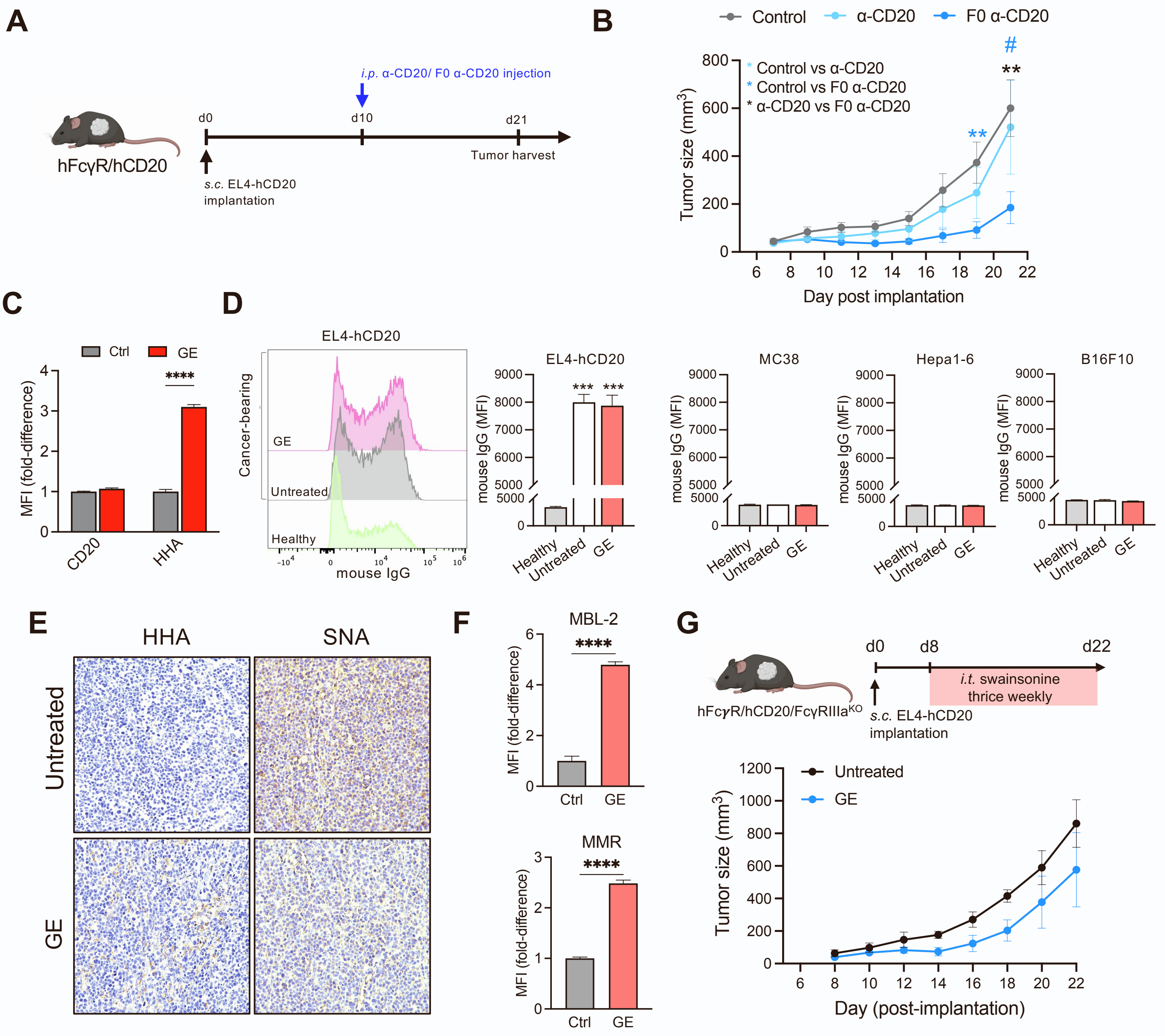

Figure S4: Impact of standard and afucosylated anti-CD20 treatment on EL4-hCD20 tumor; endogenous, murine anti-tumor IgG production; impact of glycoengineering on binding by select lectins, related to Figure 2 (A) Schematic diagram showing EL4-hCD20 cells were subcutaneously (s.c.) implanted in hFcγR/hCD20 mice, followed by α-CD20 or F0 α-CD20 treatment through intraperitoneal (i.p.) injection on day 10 and (B) Tumor growth over time. (C) hCD20 expression and high-mannose expression (HHA binding) on EL4-hCD20 cells treated in vitro with swainsonine for 24 hours. (D) Serum was collected from cancer-bearing mice and the level of murine endogenous IgG specific to EL4-hCD20 cells and other irrelevant cancer cell lines MC38 (adenocarcinoma), Hepa 1-6 (hepatocellular carcinoma) and B16F10 (melanoma) were determined. (E) Representative picture of histology analysis from tumors extracted from mice that were either untreated or treated with swainsonine (GE) intratumorally, HHA and SNA represent the level of mannosylated glycans and sialylated glycans respectively. (F) MBL or MMR binding to Jurkat cells +/- GE. (G) Schematic diagram showing EL4-hCD20 cells were s.c. implanted in hFcγR/hCD20/FcγRIIIa^{KO} mice, followed by intratumoral administration of swainsonine thrice weekly. Due to the limited availability of the

hFcγR/hCD20/FcγRIIIaKO mice, a smaller group size was used here relative to other experiments in this study (*n* = 4-6 here vs. *n* = 8-10 in other experiments), which may have precluded a significant difference between groups. The data are represented as mean +/-SEM in (B-D, F-G) Statistical analysis was calculated by one-way ANOVA in (B, D) and two-tailed unpaired student's T test in (C, F and G). $^{*}p < 0.05$, $^{**}p < 0.01$, $^{***}p < 0.001$.

Figure S5

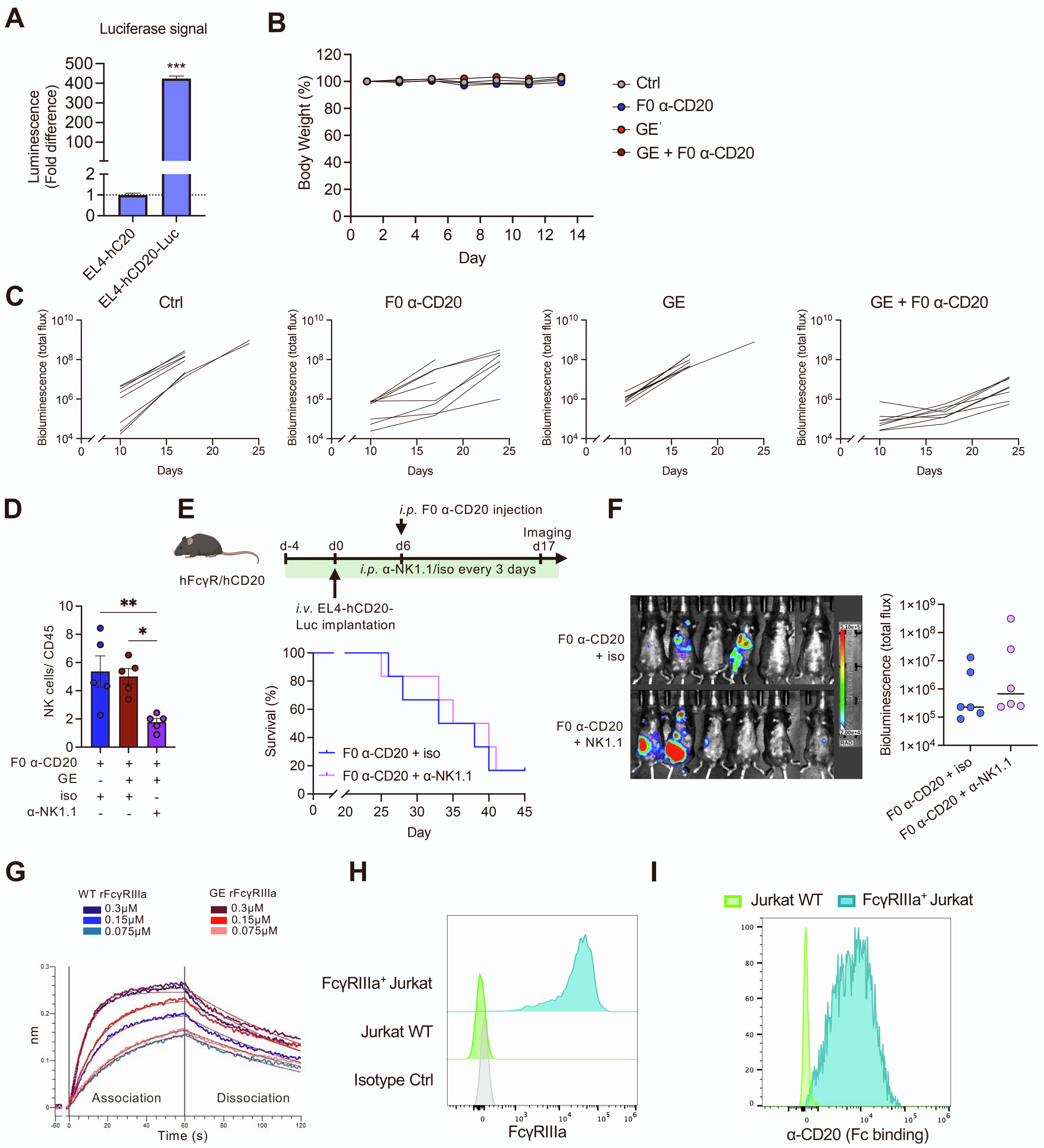

Figure S5: NK cells are dispensable for the F0 α-CD20-mediated cancer clearance in the absence of glycoengineering, related to Figure 3-5 (A) Parental and EL4-hCD20-Luc were analyzed for their luminescence post-stable transfection. (B) Body weight monitoring of hFcγR/hCD20 bearing cancer established by intravenous delivery of EL4-hCD20-Luc cells and treated with swainsonine (GE), F0 α-CD20 or a combination of both. (C) Spider-plots showing the cancer burden of mice as determined by the bioluminescence emitted on days 10, 17 and 24. Each line represents one mouse and the line breaks off if the mouse died before that measurement date.

(D) NK cells frequency in hFcγR/hCD20 mice receiving either isotype control antibody (iso) or NK cell depleting antibodies (α-NK1.1). (E) Schematic diagram showing hematologic lymphoma model established in hFcγR/hCD20 mice by intravenous delivery of EL4-hCD20-Luc, in conjunction of either iso or α-NK1.1 treatment with F0 α-CD20 treatment, and their survival (bottom) and (F) cancer burden as determined by the bioluminescence emitted on day 17. (G) Representative association and dissociation curves from the BLI-based binding affinity assay, with F0 α-CD20 immobilized on the BLI probe and WT or GE rFcγRIIIa in solution. (H) The expression level of FcγRIIIa on parental (WT) or FcγRIIIa+ Jurkat cells used in the cell-based binding assay. (I) The binding of fluorochrome-conjugated IgG (α-CD20) on either WT or FcγRIIIa+ Jurkat cells, showing no binding in the absence of cell surface FcγRIIIa. The data are represented as mean +/-SEM in (A, B). Statistical significance was calculated by two-tailed unpaired student's t test in (A, F), one-way ANOVA in (B, D) and by log-rank Mantel-Cox test in (E). $^{*}p < 0.05$, $^{**}p < 0.01$, $^{***}p < 0.001$

Supplemental references:

[S1]. Pushel, I., Mamidi, T.K., Yoo, B., Lansdon, L.A., Louiselle, D., Gibson, M., Guest, E., Wood, N.M., August, K.J., Flatt, T.G., et al. (2025). A comprehensive bulk and single-cell transcriptional atlas of pediatric leukemias. bioRxiv, 2025.2012.2023.696262. 10.64898/2025.12.23.696262.
